# A computational approach to design a polyvalent vaccine against human respiratory syncytial virus

**DOI:** 10.1038/s41598-023-35309-y

**Published:** 2023-06-15

**Authors:** Abu Tayab Moin, Md. Asad Ullah, Rajesh B. Patil, Nairita Ahsan Faruqui, Yusha Araf, Sowmen Das, Khaza Md. Kapil Uddin, Md. Shakhawat Hossain, Md. Faruque Miah, Mohammad Ali Moni, Dil Umme Salma Chowdhury, Saiful Islam

**Affiliations:** 1 Department of Genetic Engineering and Biotechnology, Faculty of Biological Sciences, University of Chittagong, Chattogram, Bangladesh; 2 Department of Biotechnology and Genetic Engineering, Faculty of Biological Sciences, Jahangirnagar University, Savar, Dhaka, Bangladesh; 3 Department of Pharmaceutical Chemistry, Sinhgad Technical Education Society’s, Sinhgad College of Pharmacy, Pune, Maharashtra India; 4 Biotechnology Program, Department of Mathematics and Natural Sciences, School of Data and Sciences, BRAC University, Dhaka, Bangladesh; 5 Department of Genetic Engineering and Biotechnology, School of Life Sciences, Shahjalal University of Science and Technology, Sylhet, Bangladesh; 6 Department of Computer Science and Engineering, School of Physical Sciences, Shahjalal University of Science and Technology, Sylhet, Bangladesh; 7 Bone Biology Division, The Garvan Institute of Medical Research, Darlinghurst, New South Wales, Australia; 8 WHO Collaborating Centre on eHealth, UNSW Digital Health, School of Public Health and Community Medicine, Faculty of Medicine, UNSW Sydney, Sydney, Australia; 9 Present Address: Artificial Intelligence and Data Science, Faculty of Health and Behavioural Sciences, School of Health and Rehabilitation Sciences, The University of Queensland, Brisbane, Australia; 10 Bangladesh Council of Scientific and Industrial Research (BCSIR), Chattogram Laboratories, Chattogram, Bangladesh

**Keywords:** Structural biology, Biotechnology, Computational biology and bioinformatics, Drug discovery, Immunology, Microbiology, Molecular biology, Structural biology

## Abstract

Human Respiratory Syncytial Virus (RSV) is one of the leading causes of lower respiratory tract infections (LRTI), responsible for infecting people from all age groups—a majority of which comprises infants and children. Primarily, severe RSV infections are accountable for multitudes of deaths worldwide, predominantly of children, every year. Despite several efforts to develop a vaccine against RSV as a potential countermeasure, there has been no approved or licensed vaccine available yet, to control the RSV infection effectively. Therefore, through the utilization of immunoinformatics tools, a computational approach was taken in this study, to design a multi-epitope polyvalent vaccine against two major antigenic subtypes of RSV, RSV-A and RSV-B. Potential predictions of the T-cell and B-cell epitopes were followed by extensive tests of antigenicity, allergenicity, toxicity, conservancy, homology to human proteome, transmembrane topology, and cytokine-inducing ability. The peptide vaccine was modeled, refined, and validated. Molecular docking analysis with specific Toll-like receptors (TLRs) revealed excellent interactions with suitable global binding energies. Additionally, molecular dynamics (MD) simulation ensured the stability of the docking interactions between the vaccine and TLRs. Mechanistic approaches to imitate and predict the potential immune response generated by the administration of vaccines were determined through immune simulations. Subsequent mass production of the vaccine peptide was evaluated; however, there remains a necessity for further in vitro and in vivo experiments to validate its efficacy against RSV infections.

## Introduction

The Human Respiratory Syncytial Virus (hRSV), a member of the family of *Paramyxoviridae,* is known to be the primary cause of lower respiratory tract infections (LRTI), including pneumonia and bronchitis—in infants, children, as well as elderly and immunocompromised individuals^1,2^. RSV is an enveloped virus that contains a single-stranded, negative-sense RNA with a genome size of about 15.2 kb. As of yet, two major RSV antigenic subtypes have been identified, RSV-A and RSV-B, exhibiting differential sequence divergence throughout their genome; RSV-A has been seen to be more prevalent than RSV-B^2,3^. However, the two subtypes can coexist and thrive, owing to RSV reinfections being common throughout the life of an infected individual, indicating that cross-immunity against distinct strains is only partial^4^. RSV severely affects immunocompromised infants as well as the geriatric population with weakened immune systems. The implicated virus infection is considered globally to be the second largest cause of death, in children under one year of age. RSV-associated acute LRTI is responsible for around 33 million serious respiratory infections globally every year, according to the World Health Organization (WHO); resulting in more than 3 million hospitalizations and about 60,000 deaths of children under 5 years of age, and 6.7% of all deaths in infants younger than a year-old. RSV infects the cells, lining the human respiratory pathway, including the ciliated epithelial cells, and causes upper and lower respiratory tract complications. Influenza-like diseases and LRTI display clinical symptoms of serious RSV infection^2,5^. Consequently, over the past two decades, RSV has become a major focus for vaccination studies to decrease the morbidity of lower respiratory tract infections. Despite the failure of the first RSV vaccine trial in 1960, many vaccines have been developed and investigated, but no one is licensed yet for commercial use^6,7^. In addition to vaccine development approaches, the use of RSV anti-infective drugs as a palliative treatment option is being explored as a potential strategy to fight against RSV infections^8^. Merely two approved RSV antivirals are currently available, which include, palivizumab, a humanized preventive monoclonal antibody, and aerosolized ribavirin for therapy. While these two antivirals can help alleviate the symptoms of RSV infections, they are not ideal for prophylactic use. Furthermore, the efficacy of ribavirin in improving outcomes for severe RSV patients remains uncertain^9,10^. Hence, the available research highlights the urgent need to develop effective RSV vaccines to successfully combat the infections caused by the virus.

In this study, an immunoinformatics approach was used to generate a polyvalent epitope-based vaccine blueprint capable of inducing a substantial immune response against both RSV-A and RSV-B types. We have targeted four highly virulent proteins for designing the subunit vaccine such as phosphoprotein (P protein), nucleoprotein (N protein), fusion glycoprotein (F protein), and major surface glycoprotein (mG protein). The F protein mediates the fusion and attachment of the virus to its target cells along with the mG protein, thus facilitating viral entry^11^. Whereas, N protein surrounds the viral genome of RSV, and the P protein is a vital component of the viral RNA-dependent RNA polymerase complex which is necessary for the proper replication and transcription of RSV^12^. Hence, this study aimed to identify potential epitopes from the P, N, F, and mG proteins of RSV, with the goal of designing a polyvalent vaccine capable of stimulating an immune response to combat RSV infection.

## Results

### Protein sequences identification and retrieval

From the NCBI database, the RSV viral strains and the query proteins were identified. Following that, the four RSV-A and RSV-B Query Proteins including P protein, N protein, F protein, and mG, were retrieved from the UniProt online database. The UniProt Accession Number and the length of the query proteins are listed in Table 1.

**Table 1:** List of targeted proteins with their UniProt accession numbers.

Strain type	Protein	UniProt accession number	Sequence length
RSV-A	P protein	P03421	241
	N protein	P03418	391
	F protein	P03420	574
	mG protein	P03423	298
RSV-B	P protein	O42062	241
	N protein	O42053	391
	F protein	O36634	574
	mG protein	O36633	299

### Epitope prediction and sorting the most promising epitopes

All of the selected proteins were found to be antigenic in the initial antigenicity analysis and the biophysical property analyses of the query proteins are listed in S1 Table. Afterwards, the RSV-A proteins were selected as models during the prediction of the T-cell and B-cell epitopes by the IEDB server for the construction of the polyvalent vaccine, meaning that the epitopes were selected using only the RSV-A proteins and then only the fully conserved epitopes were taken therefore, the epitopes should confer immunity to the selected strains of both RSV-A and RSV-B. Based on the ranking, the top MHC Class-I or Cytotoxic T Lymphocyte (CTL) and MHC Class-II or Helper T Lymphocyte (HTL) epitopes as well as top B-cell epitopes with lengths over ten amino acids were taken into consideration for further analysis. Following this, few criteria were selected to filter the best epitopes which included high antigenicity, non-allergenicity, non-toxicity, conservancy, and human proteome non-homology. Furthermore, the cytokine (i.e., IFN-γ, IL-4, and IL-10) inducing ability of HTL epitopes was also considered to determine whether they can produce at least one of these cytokines. Finally, the epitopes that met these criteria were listed as the most promising epitopes in Table 2 and were later used for the construction of the vaccine. Also, the analysis of transmembrane topology by the TMHMM v2.0 server of the most promising epitopes revealed that PEFHGEDANNR, SFKEDPTPSDNPFS, EVAPEYRHDSPD, VFPSDEFDASISQVNEK, IPNKKPGKKTTTKPTKKPTLKTTKKDPKPQTTKSKEVPTTKP epitopes were exposed outside.

**Table 2:** List of the epitopes eventually selected for the construction of the vaccine.

Name of the protein	MHC class-I epitopes	MHC class-II epitopes	B cell epitopes
P protein	VSLNPTSEK	LGMLHTLVVASAGPT	PEFHGEDANNR
	QTNDNITAR	LHTLVVASAGPTSAR	EVTKESPITSNSTIINPTNETDDTAGNKPNYQRK
	–	–	SFKEDPTPSDNPFS
	–	–	RNEESEKMAKDTSDEVSLNPTSEK
N protein	CIAALVITK	EVLTLASLTTEIQIN	EVAPEYRHDSPD
	RSGLTAVIR	–	EYRGTPRNQDLYDA
	SVKNIMLGH	–	–
F protein	KTNVTLSKK	IVIIVILLSLIAVGL	VFPSDEFDASISQVNEK
	KSALLSTNK	VIIVILLSLIAVGLL	–
	IASGVAVSK	EEFYQSTCSAVSKGY	–
	KQLLPIVNK	–	–
	ITIELSNIK	–	–
	LTSKVLDLK	–	–
mG protein	TTTQTQPSK	LSILAMIISTSLIIA	TSQIKNTTPTYLTQNPQLGISPSNPSEITS
	IFIASANHK	TLSILAMIISTSLII	IPNKKPGKKTTTKPTKKPTLKTTKKDPKPQTTKSKEVPTTKPTEEPTINTT
	–	QNPQLGISPSNPSEI	SNTTGNPELTSQ

Potential epitopes of P protein, N protein, F protein and mG protein are listed in S2, S3, S4 and S5 Tables, respectively.

### Population coverage and cluster analyses of the epitopes and their MHC alleles

**Figure 1:**
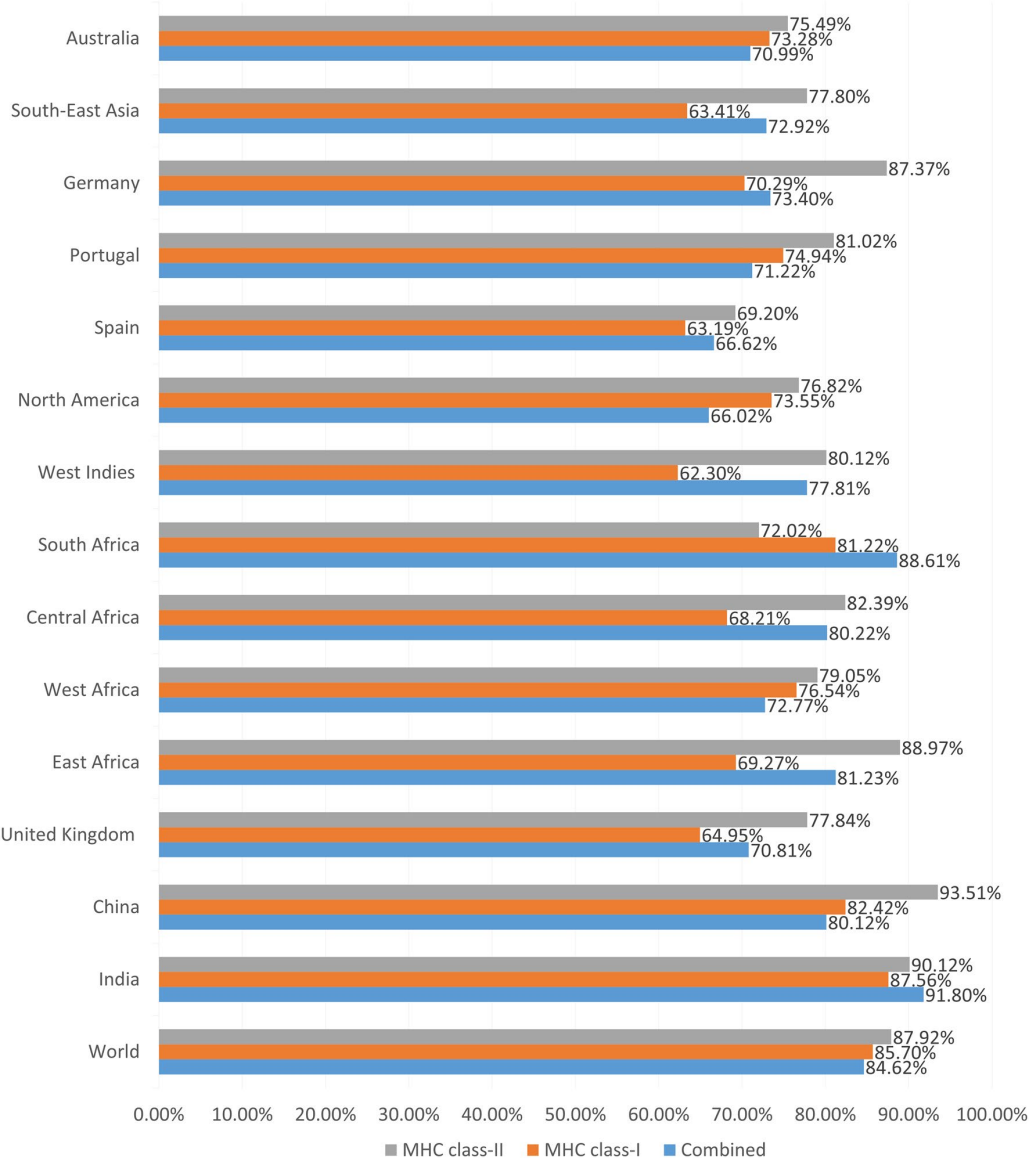
The result of the population coverage analysis of the most promising epitopes and their selected MHC alleles.

The population coverage analysis showed that 85.70% and 87.92% of the world population were covered by the MHC class-I and class-II alleles and their epitopes, respectively, and 84.62% of the world population was covered by the combined MHC class-I and class-II. While India had the highest percentage of population coverage for the CTL epitopes (87.56%) as well as HTL epitopes (93.51%), China had the highest percentage of population coverage for CTL and HTL epitopes in combination (91.80%) (Fig. 1). On the other hand, Cluster analysis of the potential alleles of MHC class I and MHC class II that may interfere with the predicted epitopes of the RSV query proteins was also conducted. S1 Fig shows the heat maps and tree maps representing the clusters. The heat map represents the strength of interaction between the alleles, with the red zone indicating stronger interactions and the yellow zone indicating weaker interactions. On the other hand, the tree map shows the hierarchical clustering of the alleles, with larger clusters represented by larger rectangles and smaller clusters by smaller rectangles. Together, these visualizations provide insight into the relationships between MHC alleles and can aid in understanding the genetic diversity of a population.

### Vaccine construction and biophysical property analyses

**Figure 2:**
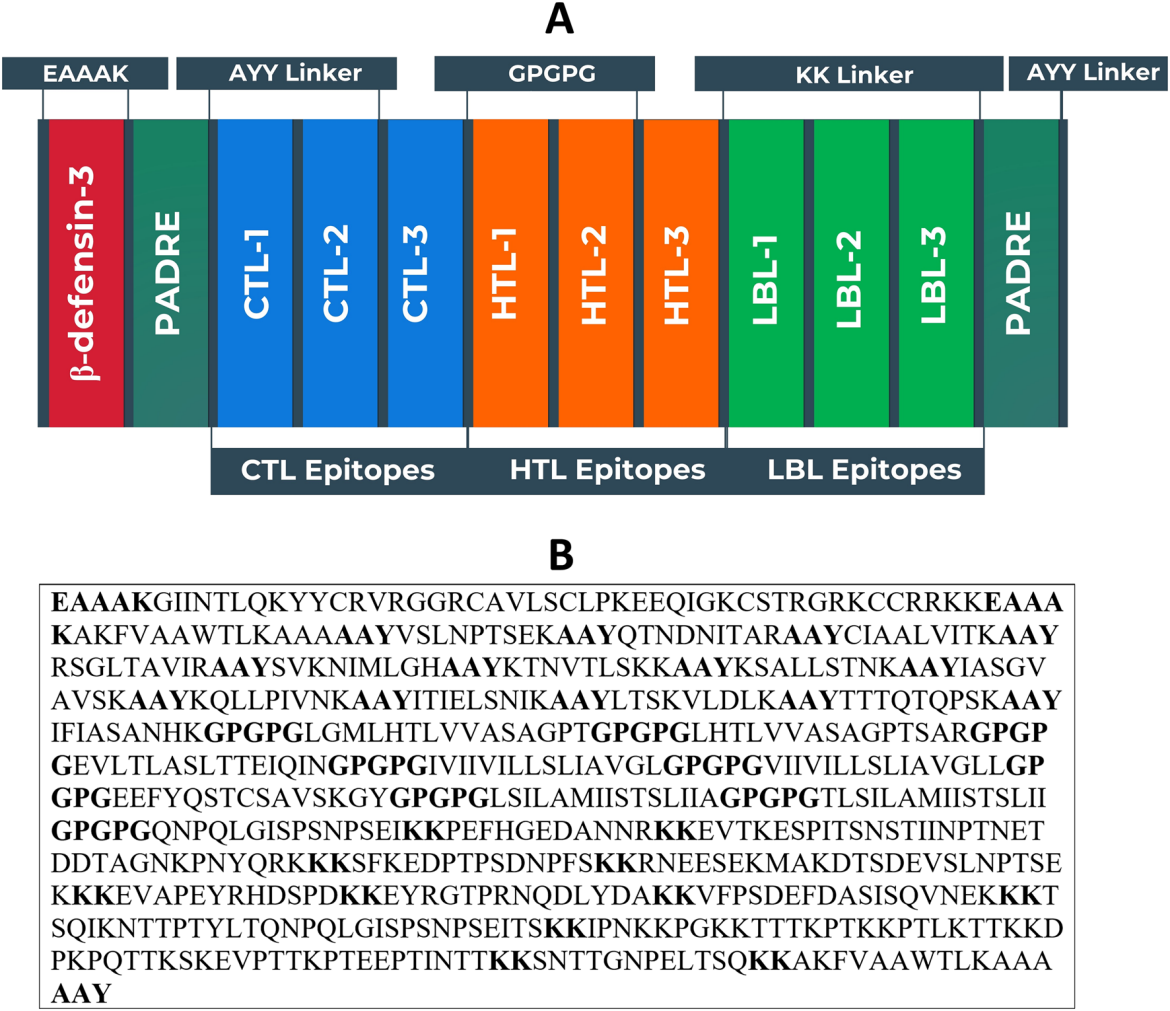
Designing of the vaccine construct. (**A**) Schematic representation of the potential vaccine construct with linkers (EAAAK, AAY, GPGPG, and KK), PADRE sequence, adjuvant (hBD-3) and epitopes (CTL, HTL, and LBL) in a sequential and appropriate manner. (**B**) Sequence of the vaccine protein. The letters in bold represent the linker sequences.

The vaccine sequence was constructed by linking the most promising T-cell and B-cell epitopes with appropriate linkers. Additionally, hBD-3 adjuvant and PADRE sequence were conjugated in the appropriate positions. A schematic presentation of the vaccine sequence is illustrated in Fig. 2. Later, during the biophysical property analyses of the vaccine, it was observed that the vaccine possessed potent antigenic properties and was non-allergenic. The vaccine had a high theoretical (basic) pI of 9.75. It had a reasonably adequate half-life in mammalian cells of 30 h and of more than 10 h in the *E. coli* cell culture system. The GRAVY value of the vaccine was considered to be significantly negative at -0.362. Additionally, both servers, Sol-Pro and Protein-sol, have also shown that the vaccine protein is soluble, attesting to its negative value. The instability index of the protein was found to be less than 40 (27.55), indicating the vaccine to be quite stable. The extinction coefficient and the aliphatic index of the vaccine were also found to be high with values, 45,770 M^−1^ cm^−1^ and 80.85, respectively.

### Vaccine structure prediction, refinement and validation

**Figure 3:**
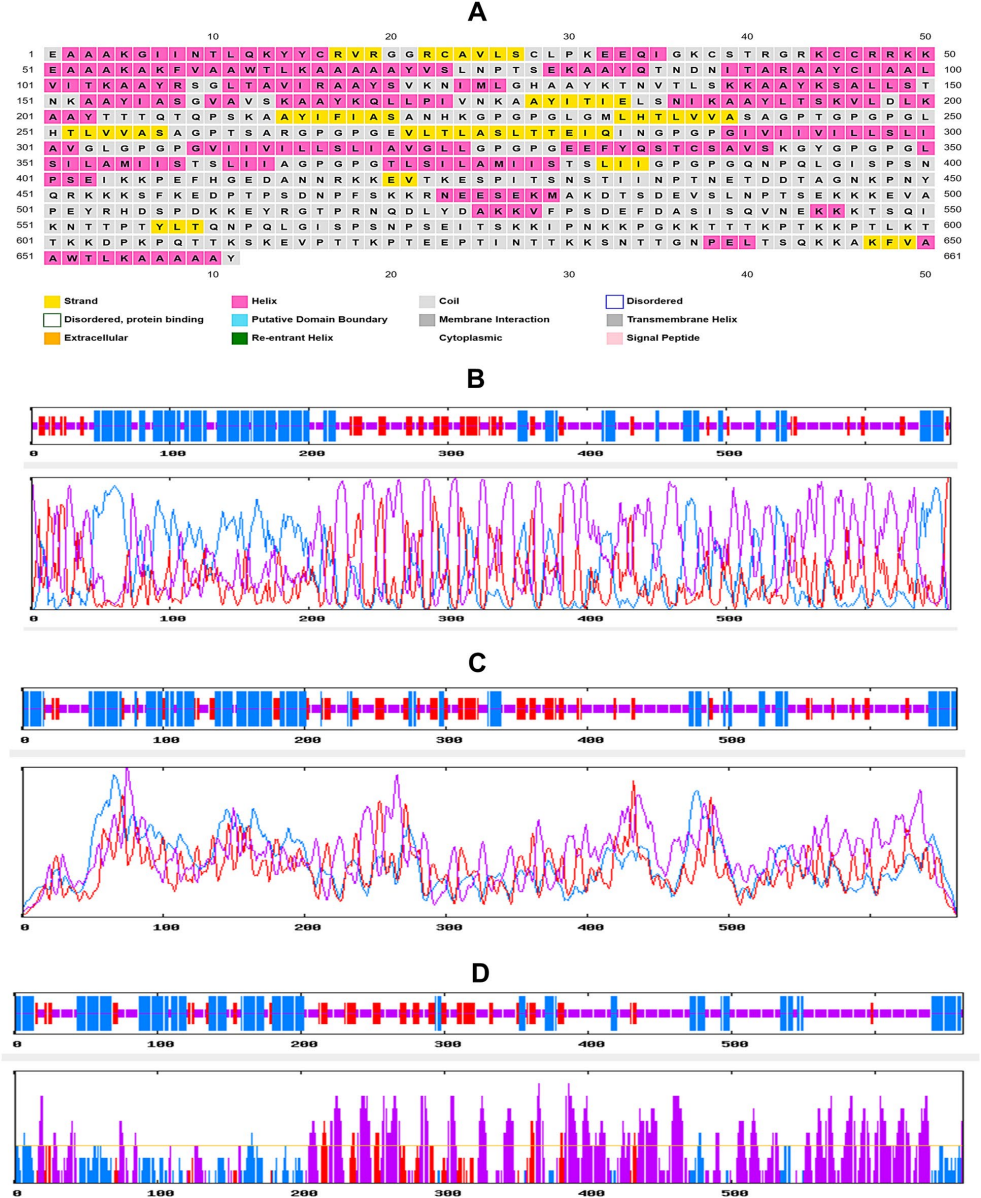
The results of the secondary structure prediction of the vaccine. (**A**) PRISPRED prediction, (**B**) GOR IV prediction, (**C**) SOPMA prediction, (**D**) SIMPA96 prediction.

The secondary structure of the vaccine protein was analyzed, revealing that the coil structure had the largest number of amino acids, while the β-strand showed the lowest percentage. Predictions from four different servers, as depicted in Fig. 3, were compared and the amino acid percentages of α-helix, β-strand, and coil structure were listed in Table 3. The servers showed similar predictions, and the adjuvant appeared to generate potential variations in the vaccine protein's secondary structure.

**Table 3:** Results of the secondary structure analysis of the vaccine construct.

Secondary structure elements	PRISPRED	GOR IV	SOPMA	SIMPA96
α-helix	33.51%	32.98%	30.56%	29.61%
β-strand	16.22%	15.58%	18.46%	14.50%
Coil structure	50.25%	51.44%	50.98%	55.74%

**Figure 4:**
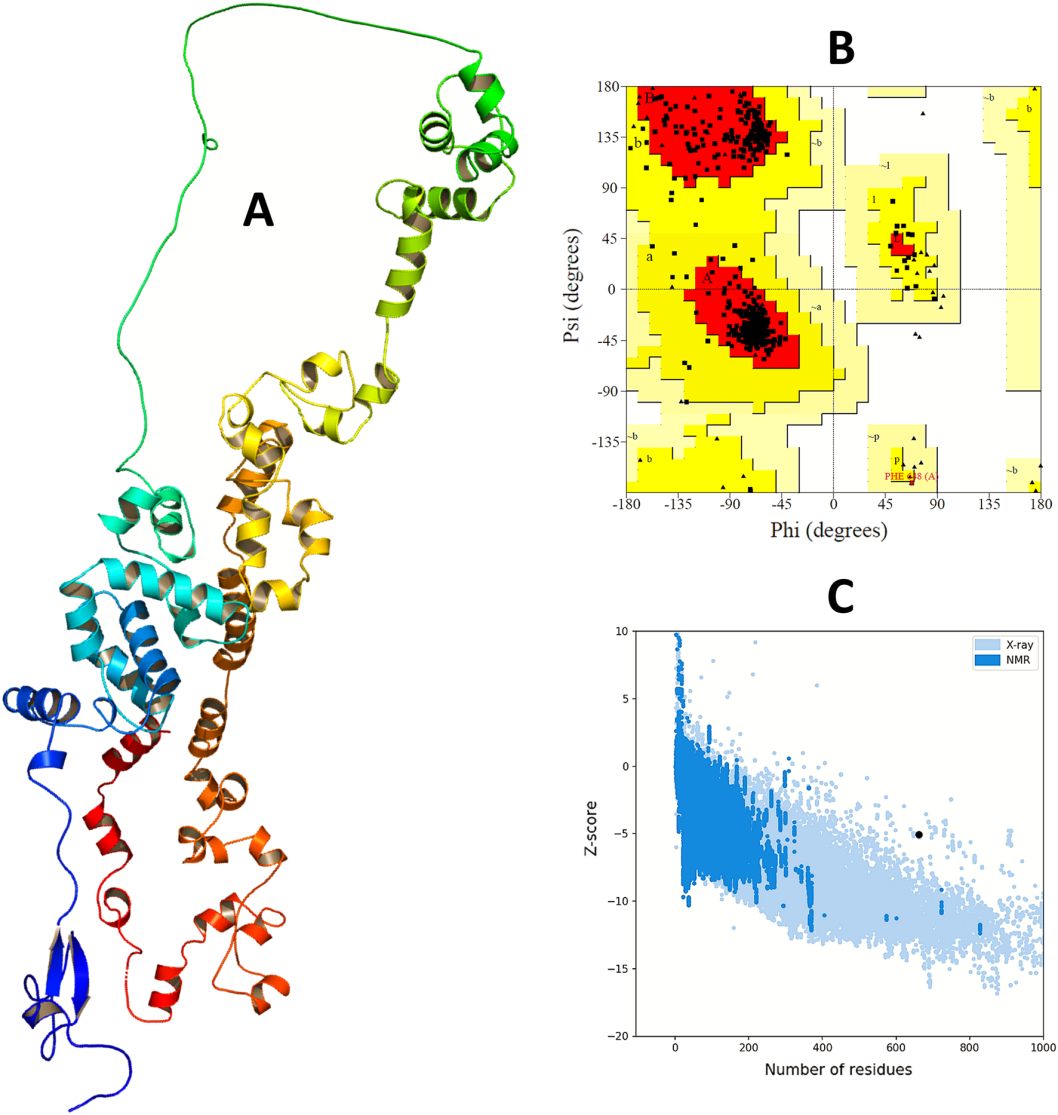
Prediction, refinement and validation of the tertiary structure of vaccine. (**A**) The tertiary or 3D structure of the vaccine construct modelled, refined and visualized by RaptorX, GalaxyWEB server, and BIOVIA Discovery Studio Visualizer v. 17.2 respectively. (**B**) The results of the Ramachandran plot analysis generated by PROCHECK server and (**C**) quality score or z-score graph generated by the ProSA-web server of the refined vaccine construct. In the Ramachandran plots, the orange and deep yellow coloured regions are the allowed regions, the light yellow regions are the generously allowed regions and the white regions are the outlier regions and the glycine residues are represented as triangles.

A 3D model of the vaccine was generated using the RaptorX online server, which had a significantly good quality with a low p-value of 8.71e-05 in four domains. Homology modeling was performed using 1KJ6A as a template from the Protein Data Bank. Additionally, the vaccine structure was modeled using Modeller to further improve its quality, as shown in Fig. 4.

Afterwards, the modelled structure of the vaccine was refined and validated using the Ramachandran plot and z-score plot. The Ramachandran plot revealed that 93.5% of the amino acids in the vaccine protein were in the most preferred region, while 6.3% were in additional approved regions, 0.2% in the generously permitted regions, and no residues in the disallowed regions. The z-score of the modelled vaccine was − 5.08. Overall, the protein validation analyses revealed a reasonably consistent structure in the refined form, as shown in Fig. 4. The structure was deemed suitable for further molecular docking and simulation studies**.**

### Analysis of conformational B-lymphocytic epitopes and disulfide engineering of vaccine

**Figure 5:**
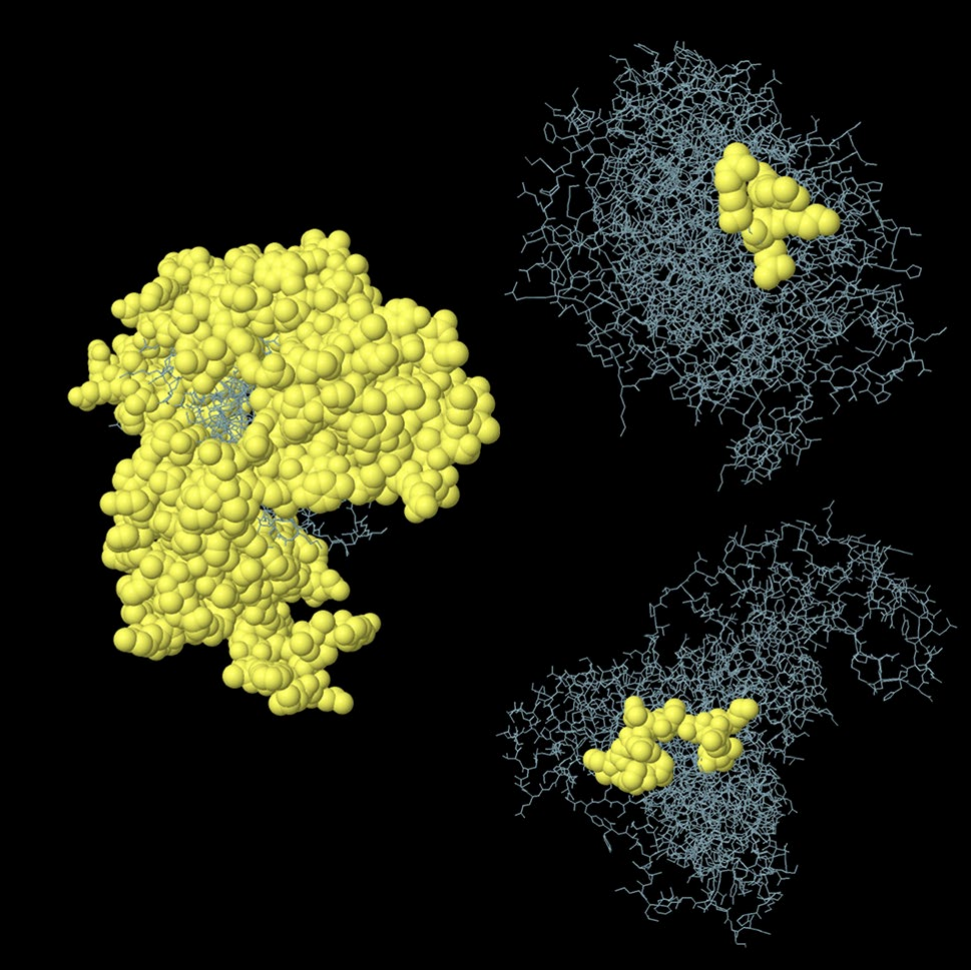
Graphical representations of the predicted conformational B-cell epitopes of the modelled vaccine indicated by yellow coloured ball-shaped structures.

The ElliPro server was used to predict the conformational B-cell epitopes of the vaccine protein. The analysis revealed three non-contiguous B-cell epitopes, encompassing a total of 333 amino acid residues with values ranging from 0.506 to 0.675. The conformational epitopes varied in size, ranging from 4 to 394 residues. The three-dimensional representation of the conformational B-cell epitopes of the designed multi-epitope-based RSV vaccine and the epitope residues are presented in Fig. 5 and listed in S6 Table. The disulfide bonds of the vaccine structure were predicted using the DbD2 server for disulfide engineering analysis. Three pairs of amino acids with bond energy below 2.2 kcal/mol were selected: 23 Cys and 38 Cys, 414 Ala and 414 Lys, and 513 Tyr and 516 Thr. The selected pairs of amino acids were used to form the mutant vaccine in the DbD2 server, which contains potential disulfide bonds within (S2 Fig).

### Post-translational modification analysis

In the post-translational modification analysis of the vaccine, Four N-glycosylation sites and sixty-three O-glycosylation sites were predicted in the vaccine construct sequence. The findings suggest that a significant amount of glycosylation may have occurred inside the predicted vaccine construct, which might improve the vaccine's efficacy and immunogenicity. In addition, the vaccine protein sequence has ninety-six phosphorylated residues (i.e., serine residues (S), threonine (T), and tyrosine (Y) phosphorylation sites) according to the NetPhos v2.0 server output. The server-provided plots containing the N-glycosylation sites and phosphorylation are given in S3 Fig.

### Analysis of protein–protein docking

Protein–protein docking analysis was performed to demonstrate the vaccine's ability to interact with various crucial molecular immune components i.e., TLRs. When docked using ClusPro 2.0, it demonstrated very high binding affinities with all its targets (TLRs). It has been further studied using the ZDOCK server where the vaccine protein also displayed very strong interaction with the TLRs. The lowest energy level obtained for docking between the vaccine construct and TLR-1, TLR-2, TLR-3, TLR-4, and TLR-9 were − 986.1, − 1236.7, − 1084.4, − 1260.8, and − 1226.4, respectively. The lowest energy level between the vaccine and TLRs indicated the highest binding affinity.

### Molecular dynamics simulation studies and MM-PBSA calculations

**Figure 6:**
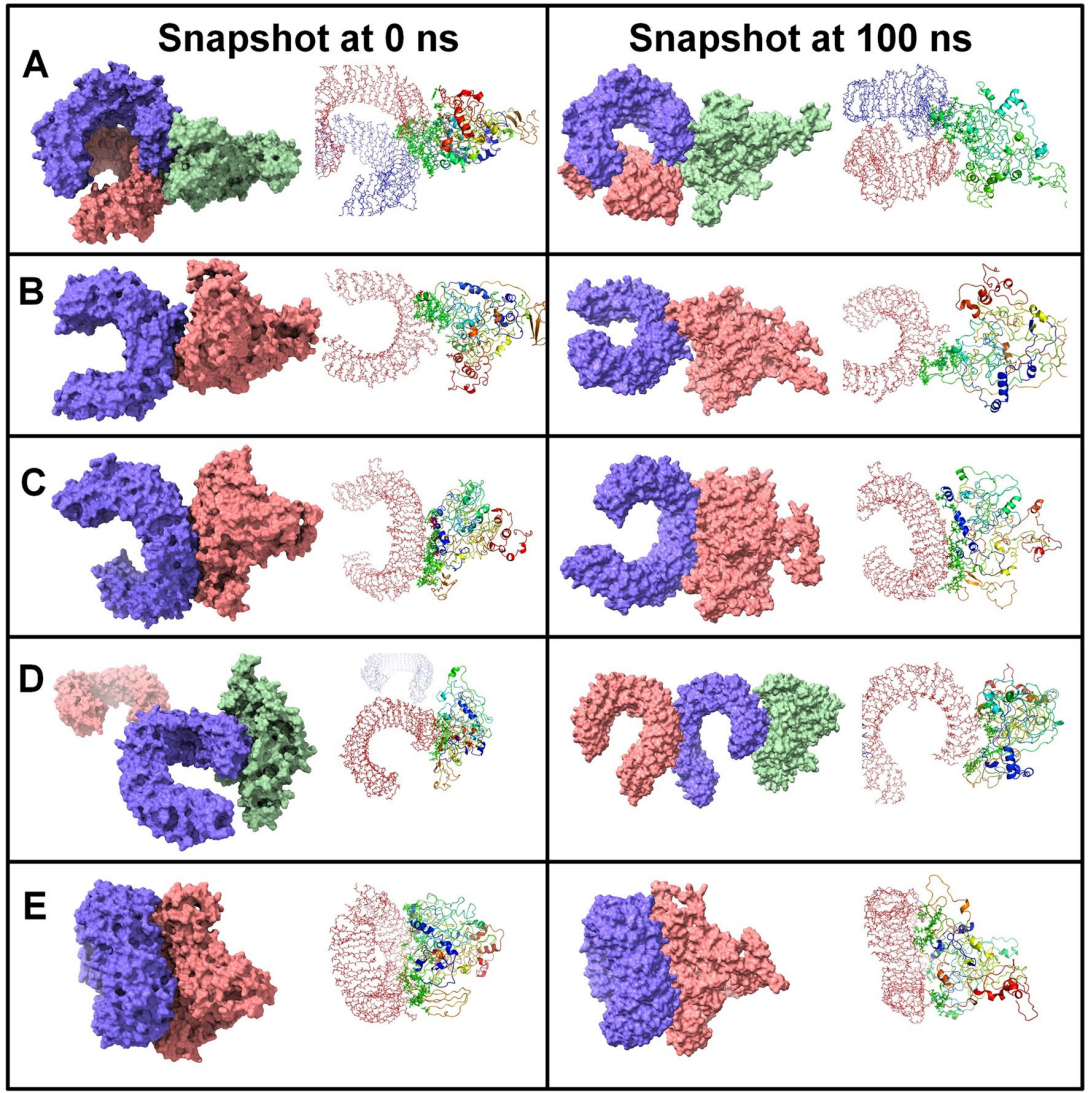
Snapshots of equilibrated (initial) systems and last trajectories. Vaccine bound complexes of (**A**) TLR1, (**B**) TLR2, (**C**) TLR3, (**D**) TLR4, and (**E**) TLR9 (For each snapshot the surface representation and cartoon representations are shown).

MD simulation is an effective method for the analysis of biological systems and it provides many mechanistic insights into the possible behavior of the system under a simulated biological environment^13^. Gromacs 2020.4 was used to carry out the production phase MD and the analysis of resulting trajectories was undertaken to understand the structural properties, and interaction between different TLRs and the predicted vaccine protein at a molecular level. The snapshots of equilibrated structures of each TLR-vaccine complex and the snapshots of the last trajectories are shown in Fig. 6. The visual inspection of trajectories at different time intervals suggested that the side chains of the vaccine make different interactions with chain A of all TLRs except TLR1, where vaccine side chains were found to be interacting with side chains of both A and B chains.

**Figure 7:**
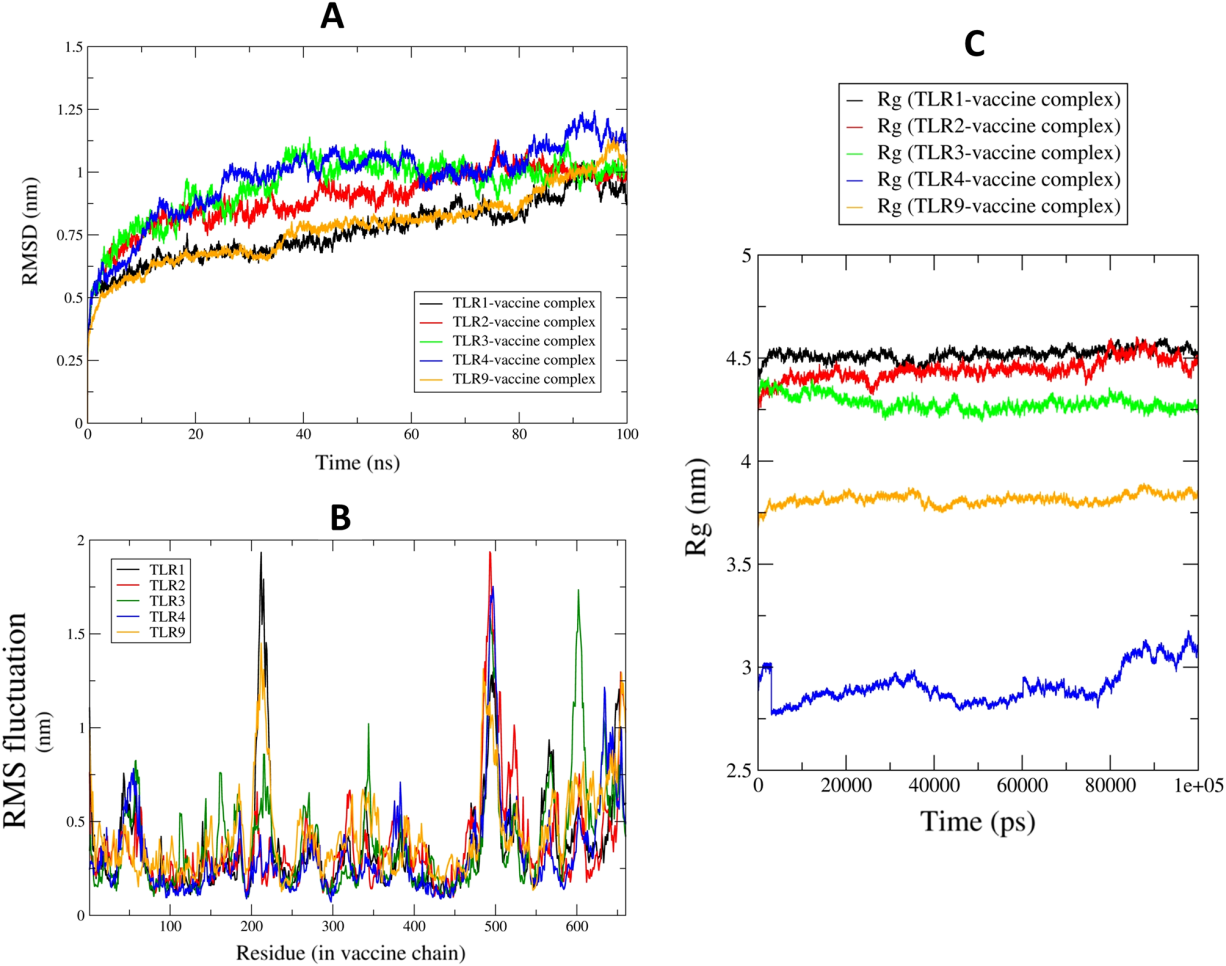
Results of the molecular dynamics simulation studies. (**A**) Root mean square deviations in the investigated systems, (**B**) Root mean square fluctuations in the side chain atoms of vaccine, and (**C**) Radius of gyration of the vaccine.

Root mean square deviations (RMSD) analysis gives insights into how the backbone atoms move relative to the initial equilibrated positions. Lower the RMSD, better the stability of the corresponding system. In the present work, we measured the RMSD in backbone atoms of entire protein–protein complexes of TLRs with a vaccine. Figure 7A shows the RMSD in the investigated systems. The evaluation of root means square fluctuations (RMSF) provides insights into the possible changes in the secondary structure of protein under investigation. In the present work, the RMSF in the side chain atoms of residues in each system was measured. As the TLR-vaccine systems have multiple chains, the RMSF evaluation is performed on each chain of the complex to understand which residues are involved in the key contacts. The RMSF in the TLR side-chain atoms is shown in Fig. 7B. The RMSF in other chains in each of the TLRs is given in S4 Fig. The analysis of radius of gyration (Rg) provides the overall measurement of compactness of the system^14^. The results of total Rg are shown in Fig. 7C. Analysis of non-bonded interactions such as hydrogen bonds is quite challenging in protein–protein complexes. The side chains of the proteins participate in hydrogen bond interactions. The hydrogen bond analysis of all the trajectories was performed with the h-bond module of Gromacs, while the key residues at the interface of the TLR chain and vaccine chain were analyzed through the chimeraX program^15^. The results of the hydrogen bond analysis are shown in S5 Fig.

### Immune simulation studies

**Figure 8:**
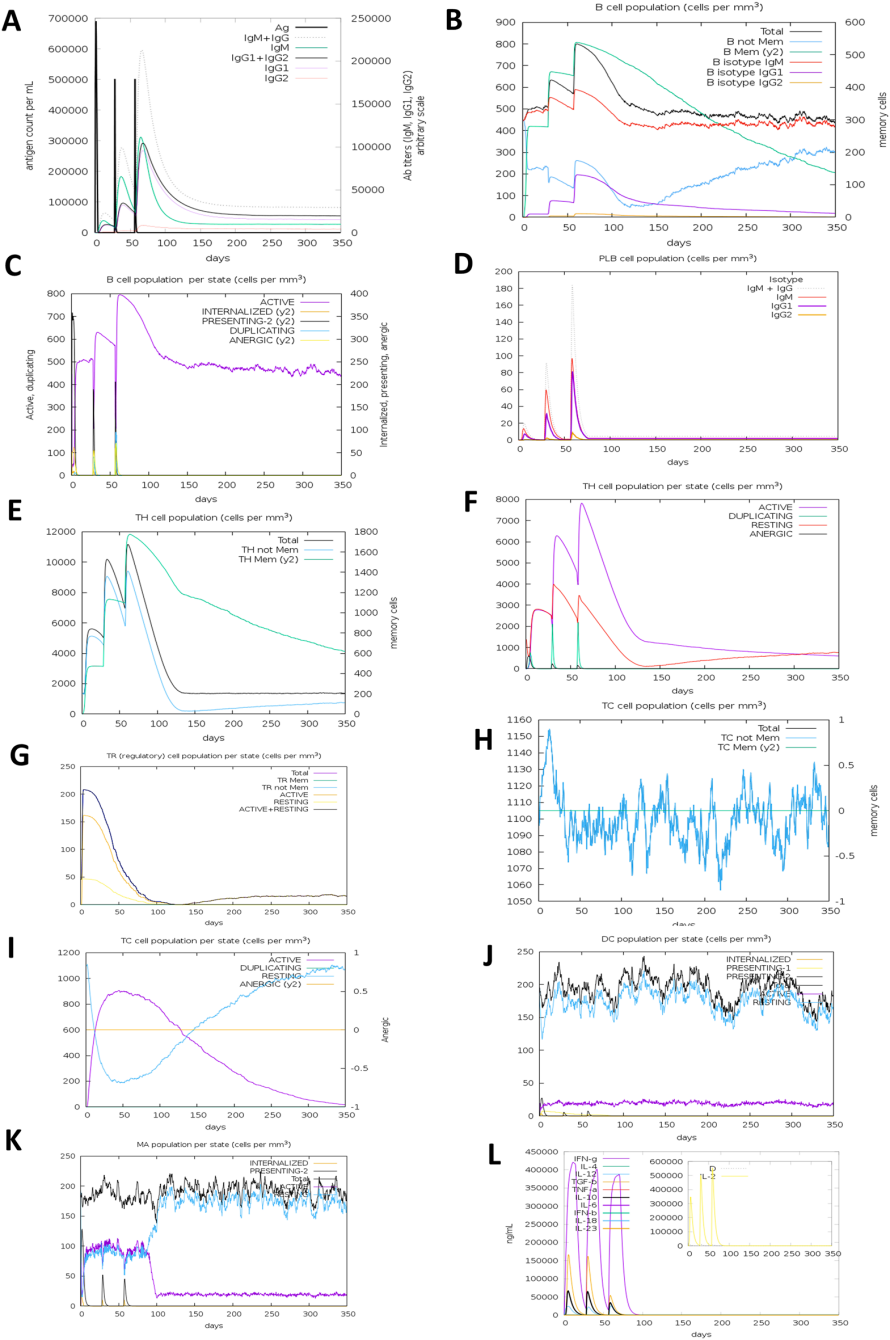
C-IMMSIMM representation of the immune simulation of the designed vaccine construct. (**A**) The immunoglobulin and immunocomplex response to the vaccine inoculations (lines hued in black) and specific subclasses are indicated by coloured lines, (**B**,**C**) elevation of the B-cell population throughout the three injections, (**D**) rise in the plasma B-cell population throughout the injections, (**E**,**F**) elevation of the helper T-cell population throughout the three injections, (**G**) decrease in the regulatory T lymphocyte concentration throughout the three injections, (**H**,**I**) augmentation in the cytotoxic T lymphocyte population throughout the injections, (**J**,**K**) augmentation in the population of active dendritic cell and macrophage respectively, per state throughout the three injections, (**L**) Rise in the concentration of different types of cytokines throughout the three injections.

The immune simulation study of the designed vaccine was conducted using the C-ImmSimm server which forecasts the activation of adaptive immunity as well as the immune interactions of the epitopes with their specific targets. The analysis exhibited that the primary immune reaction to the vaccine could be stimulated substantially after administration of the vaccine, as demonstrated by a steady rise in the levels of different immunoglobulins i.e., (IgG1 + IgG2, and IgG + IgM antibodies) (Fig. 8A). It was also expected that the concentrations of active B cells (Fig. 8B,C), plasma B cells (Fig. 8D), helper T cells (Fig. 8E,F), and cytotoxic T cells (Fig. 8H,I) could steadily increase, reflecting the vaccine's capacity to create a very high secondary immune response and healthy immune memory. However, Fig. 8G demonstrates that the concentration of regulatory T cells would gradually decrease throughout the phases of the injections, which represents the decrease in suppression of vaccine-induced immunity by regulatory T cells. In comparison, the rise in dendritic cell and macrophage concentrations showed that these APCs had a competent presentation of antigen (Fig. 8J,K). The simulation result also predicted that the constructed vaccine could generate numerous forms of cytokines, including IFN-γ, IL-23, IL-10, and IFN-β; some of the most critical cytokines for producing an immune response to viral infections (Fig. 8L). Therefore, the overall immune simulation analysis showed that after administration, the proposed polyvalent multi-epitope vaccine would be able to elicit a robust immunogenic response.

### Codon adaptation, in silico cloning, and interpretation of the vaccine mRNA secondary structure

**Figure 9:**
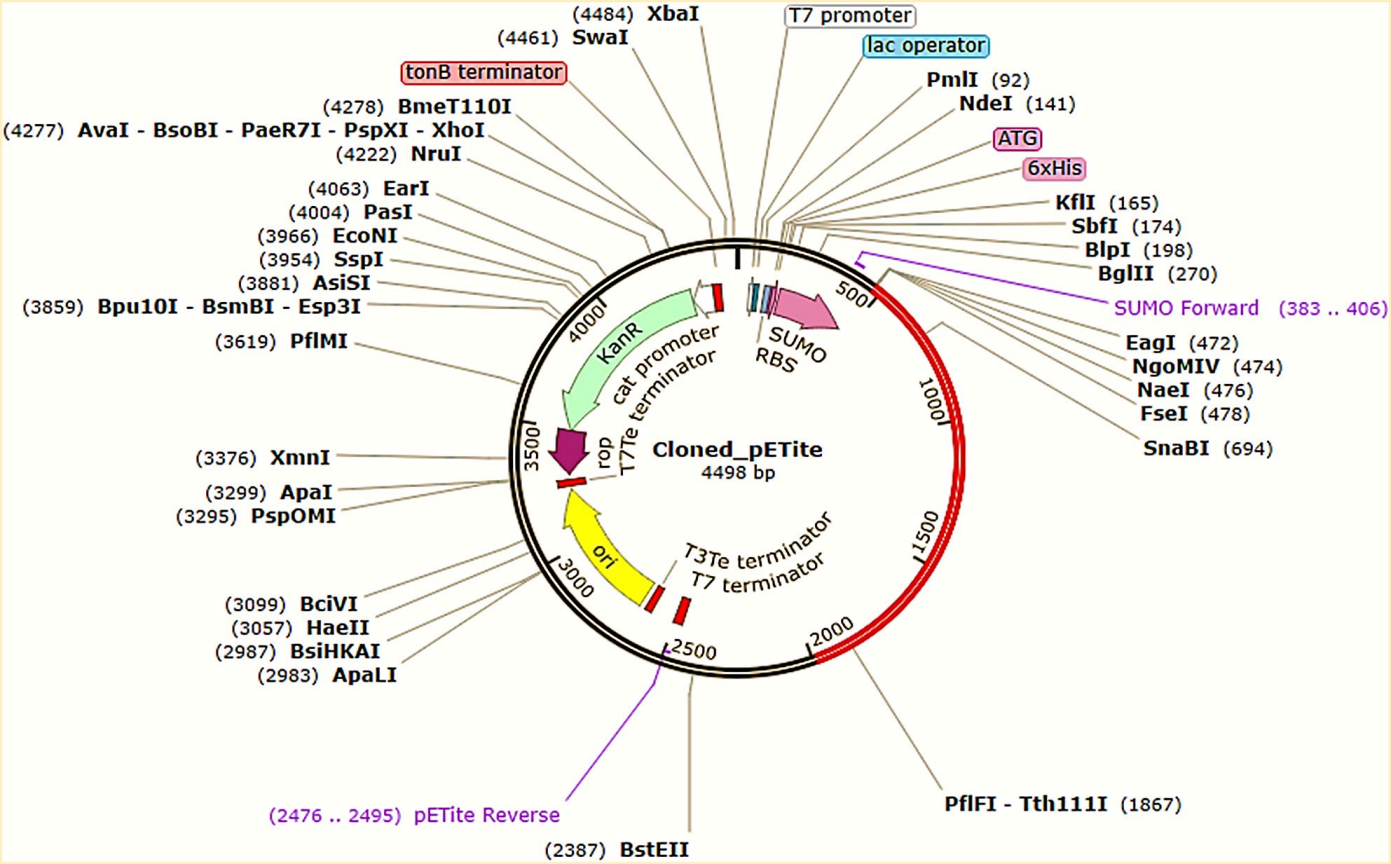
In-silico cloning of the vaccine sequence in the pETite plasmid vector. The codon sequence of the final vaccine is presented in red generated by the JCat server. The pETite expression vector is in black.

The protein sequence of the vaccine was optimized for in-silico cloning and plasmid construction using the JCat server. The Codon Adaptation Index (CAI) value was 0.98, and the GC content was 50.23%, which falls within the desired range. The sequence was graphically illustrated after codon adaptation, as shown in S6 Fig. The codon-adapted vaccine DNA sequence was then inserted between the EaeI and StyI restriction sites into the pETite vector plasmid. The resulting recombinant plasmid was named "Cloned_pETite" (Fig. 9). Later, the secondary structure of the vaccine mRNA was predicted using the Mfold and RNAfold servers. The minimum free energy scores predicted by the Mfold and RNAfold servers were − 549.30 kcal/mol and − 526.30 kcal/mol, respectively. These scores were consistent with each other. The vaccine mRNA secondary structure is depicted in S7 Fig.

## Discussion

Our study used immunoinformatics techniques to design a polyvalent epitope-based vaccine against RSV A and B subtypes by targeting four antigenic proteins of the viruses. The proteins' antigenicity and biophysical properties were predicted and found to be antigenic, with high aliphatic index, extinction coefficient, and negative GRAVY values, suggesting their stability, light-absorption ability, and hydrophilic nature. The potential T-cell and B-cell epitopes of the selected proteins, crucial in activating cytotoxic T-cells, helper T-cells, and B-cells to generate an effective immune response^16^, were analyzed in our study. To identify potential epitopes that can elicit an immune response in individuals with diverse human leukocyte antigen (HLA) genotypes, epitope prediction for multiple alleles was considered. The high polymorphism and variability in HLA allele combinations among individuals can significantly impact their ability to present and respond to specific epitopes, emphasizing the need for epitope prediction for multiple HLA alleles. This approach is essential in developing effective vaccines and immunotherapies that can target a wider population. A rigorous screening process was conducted to identify epitopes that were highly conserved, antigenic, non-allergenic, and non-toxic. This screening process was critical in ensuring that the vaccine is safe for use and has broad-spectrum activity against both RSV-A and RSV-B viruses. Specifically, HTL epitopes that demonstrated the ability to elicit cytokine production such as IFN-γ, IL-4, or IL-10 were ultimately chosen for the development of the vaccine. These cytokines are known to play a crucial role in modulating the immune response to viral infections such as RSV^17^. IFN-γ is a pro-inflammatory cytokine that has been strongly linked to respiratory syncytial virus (RSV) pathogenesis. Studies suggest that, IFN-γ plays a dual role in the control of RSV infection by limiting viral replication and preventing airway obstruction^18^. In contrast, IL-10 is an anti-inflammatory cytokine that has been demonstrated to regulate the immune response to viral infection by reducing the production of pro-inflammatory cytokines and limiting tissue damage caused by excessive inflammation^19,20^. IL-4 is also an anti-inflammatory cytokine that can play a crucial role in the regulation of the immune response to RSV infection by promoting Th2 cell differentiation and antibody production. It stimulates the production of immunoglobulin E (IgE) and Th2 cytokines, such as IL-5 and IL-13, which can help in clearing the virus and preventing further infection^21^. Therefore, cytokines like IFN-γ, IL-10, and IL-4 were given special consideration during the selection of HTL epitopes for the vaccine, since they may play a crucial role in mediating communication between various immune cells after the vaccine is administered^22^. In the MHC cluster analysis, the result presented indicates that the MHC Class-I and Class-II alleles and their epitopes, when considered separately, have a population coverage of over 85% globally, which suggests that the vaccine has the potential to provide protection to a large proportion of the world's population. When the MHC Class-I and Class-II alleles and their epitopes are combined, the coverage drops slightly to 84%, still representing a significant proportion of the population. Moreover, when the selected epitopes are compared to the overall population, they exhibit a greater individual percentage coverage, which suggests that the vaccine has the potential to be effective worldwide against RSV infections. This is an important consideration when developing vaccines since diseases like RSV can affect people globally, and a vaccine that can provide broad protection across different populations can be very valuable in preventing and controlling the spread of the disease. For vaccine construction, we selected four linkers (EAAAK, AAY, GPGPG, and KK) for conjugation with the most promising RSV epitopes based on their ability to provide optimal spacing, flexibility, and stability to the conjugates. The EAAAK linker contains glutamic acid and lysine residues, AAY contains alanine and tyrosine residues, GPGPG contains glycine and proline residues, and KK contains lysine residues. These linkers were chosen to enhance solubility, flexibility, stability, and immunogenicity of the conjugates^23–25^. An innate antimicrobial peptide, hBD-3, was selected as an adjuvant to improve the antigenicity, immunogenicity, durability, and longevity of the vaccine^26^. The peptide was chosen due to its potential to induce the expression of co-stimulatory molecules on the surface of monocytes and myeloid dendritic cells, as well as its ability to prevent the fusion of the virus and stimulate various immune responses^27,28^. Additionally, the PADRE sequence was incorporated to further strengthen the immunogenic reaction of the vaccine^29^. After constructing the vaccine, its antigenicity, allergenicity, and biophysical properties were assessed, revealing the vaccine protein to be suitable for further refinement and validation processes. The results indicate that the constructed vaccine protein is a basic, hydrophilic, and stable protein with desirable biophysical properties. The high theoretical pI, negative GRAVY value, low instability index, high extinction coefficient, and aliphatic index suggest that the protein is soluble, less likely to undergo conformational changes or degradation, and highly stable. These properties make the vaccine protein a promising candidate for further modelling refinement and validation^30–33^. The secondary structure analysis of the vaccine showed a higher abundance of coiled regions and a lower amount of β-strand, indicating greater stability and conservation of the predicted vaccine model. The tertiary structure was predicted and refined, resulting in a high p-value of 8.71e−05 in four domains of the protein, indicating an accurate prediction. In an effort to increase the stability of the vaccine structure, disulfide engineering was conducted to identify potential disulfides that can increase the protein's thermal stability^34^. Following the prediction and refinement of the tertiary structure of the vaccine, an analysis of disulfide engineering and posttranslational modifications was conducted. The aim of this analysis is to provide insight for future synthesis of the vaccine, with the potential to increase the vaccine's stability and immunogenicity. Through disulfide engineering, three pairs of amino acids in the vaccine were identified which could improve protein stability and facilitate the study of protein dynamics and interactions^35^. Further, the vaccine construct was analysed for posttranslational modifications and identified four N-glycosylation sites, sixty-three O-glycosylation sites, and ninety-six phosphorylated residues. Incorporating these modifications into vaccine designs can optimize vaccine efficacy and improve protection against viral infection. Posttranslational modifications, including glycosylation, phosphorylation, and acetylation, play a significant role in vaccine effectiveness and immunological responses. Glycosylation, in particular, can enhance antigenicity and immunogenicity, while phosphorylation and acetylation can promote immune recognition and improve protein stability^36–38^. The proposed RSV vaccine was analyzed using molecular docking, which revealed a strong interaction with Toll-like receptors (TLRs) critical in RSV pathogenesis. TLR4 showed the lowest energy level with the vaccine, indicating the highest interaction, while TLR2 and TLR9 also demonstrated high interactions. TLR2, TLR1, TLR6, TLR3, and TLR4 are critical TLRs in RSV pathogenesis, with TLR2/1 and TLR2/6 complexes enhancing early innate inflammatory responses and controlling viral replication. The vaccine's strong affinity with all target TLRs suggests that it may induce a robust immune response upon administration^39–43^. Following the molecular docking analysis, MD simulation was performed to examine the molecular stability of five docked vaccine-TLR complexes in response to external forces exerted by the surrounding environment. The TLR1-vaccine complex was found to be the most stable, while the TLR4-vaccine complex had higher RMSD despite having a similar number of chains. The RMSF analysis revealed that the residues in the range 190–240 and 480–530 had large magnitudes of fluctuations in all the complexes, with TLR3-vaccine complex showing slightly larger fluctuating side chains than others. The non-bonded interaction analysis showed that the TLR1-vaccine complex had the highest number of hydrogen bonds, with around 10 being formed till around 50 ns MD time interval, which steadily lowered to around 5 hydrogen bonds till 80 ns and thereafter rose to 10 hydrogen bonds. The TLR4-vaccine complex had the lowest number of hydrogen bonds formed, around 5, compared to other systems. Overall, the study showed that the docked vaccine-TLR complexes had reasonable stability, and the TLR1-vaccine complex had the highest stability among the five complexes. Actual residues involved in the hydrogen bond formation as investigated in the last trajectory are tabulated in the S7 Table. The proposed vaccine was found to induce humoral and cell-mediated immune responses, resulting in increased memory and plasma B cells, cytotoxic and helper T cells, and various antibodies. The activation of helper T cells led to strong adaptive immunity and antigen presentation. The cytokine profile was enriched, and gradual increases in mucosal immunoglobulins were predicted, which is crucial for respiratory viruses like RSV. The vaccine also showed a potential decrease in regulatory T cell suppression of vaccine-induced immunity. Overall, the vaccine provides broad-spectrum immunity against viral invasions and stimulates the immune system predominantly at mucosal surfaces. These results suggest that the proposed vaccine construct is likely to be effective after vaccination. Finally, the study aimed to identify potential codons required for generating a recombinant plasmid that can express the vaccine in E. coli strain K12 for mass manufacturing. Codon adaptation and in silico cloning studies were performed, and the results showed significantly good results with a CAI value of 0.98 and a GC content of 50.23%, indicating the potential for high-level expression^44^. The optimized vaccine DNA sequence was inserted into the pETite plasmid vector, containing SUMO and 6X His tags that could promote purification and downstream processing of the vaccine^45^. The stability of the vaccine mRNA secondary structure was predicted using Mfold and RNAfold servers, showing a much lower minimal free energies, indicating that the predicted vaccine could be very stable upon transcription. Overall, these findings suggest that the proposed vaccine construct has the potential for mass production and could be very stable, making it a promising candidate for further development. However, further research through in vitro or in vivo studies is required to strongly verify the immunogenicity, efficacy, stability, safety, and diverse biophysical properties of the proposed vaccine.

## Methods

**Figure 10:**
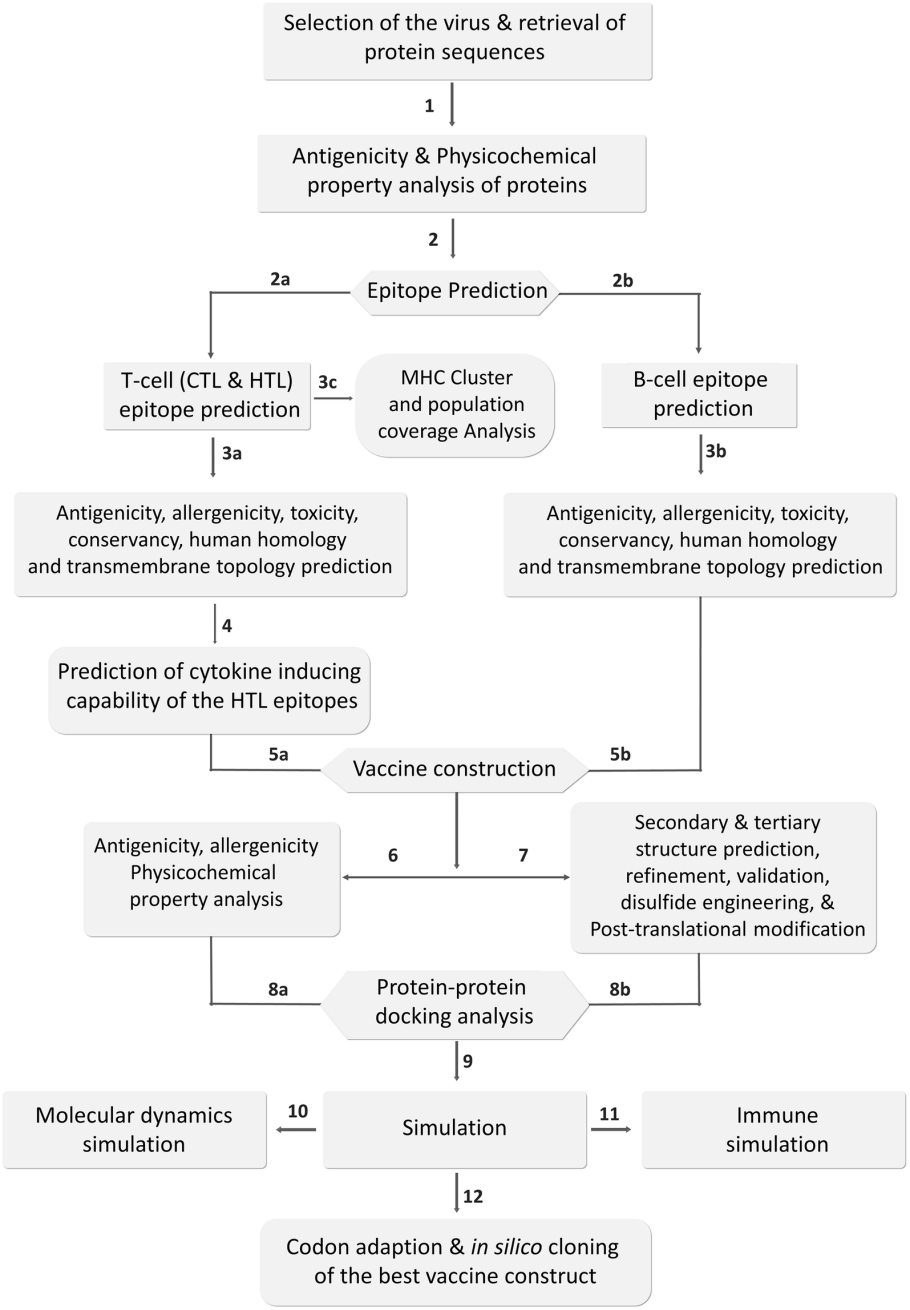
The step-by-step procedures of immunoinformatics and molecular dynamics approaches used in the vaccine designing study.

The high throughput immunoinformatics and MD approaches of vaccine designing are illustrated in a step-by-step processes in Fig. 10.

### Protein sequences identification and retrieval

Through existing literature reviews in the National Center for Biotechnology Information (NCBI) (https://www.ncbi.nlm.nih.gov/) database, the RSV-A and RSV-B viruses were identified and selected along with their target proteins (i.e., P protein, N protein, F protein, and mG protein). The sequences of target proteins of the selected strains (i.e., RSV strain A2 and RSV strain B1) were then extracted from the UniProt (https://www.uniprot.org/) database in FASTA format. The NCBI Protein database is a collection of SwissProt, PIR, PRF, and PDB sequences. It also includes GenBank, RefSeq, and TPA translations from elucidated coding regions.

### Prediction of T-cell and B-cell epitopes

Before epitopes prediction, antigenicity and biophysical properties of the target proteins were analysed. Using the online antigenicity prediction tool, VaxiJen v2.0 (http://www.ddg-pharmfac.net/vaxijen/VaxiJen/VaxiJen.html), the antigenicity of the target protein sequences was predicted with the prediction precision parameter threshold kept at 0.4^46^. Subsequently, ProtParam tool of the ExPASy server (https://web.expasy.org/protparam/) was used to determine the biophysical properties of the target proteins^47^. Afterwards, the T-cell and B-cell epitope prediction was performed using the Immune Epitope Database or IEDB (https://www.iedb.org/), which contains extensive experimental data on antibodies and epitopes^48^. For the prediction of CTL epitopes for several HLA alleles, i.e., HLA A*01:01, HLA A*03:01, HLA A*11-01, HLA A*02:01, HLA A*02:06, and HLA A*29:02, the recommended IEDB NetMHCpan 4.0 prediction method was used. The default prediction method selection of the server is ‘IEDB recommended’ which utilizes the best available technique for a specific MHC molecule based on the availability of predictors and observes the predicted performance for a specific allele. Again, for the prediction of HTL epitopes for DRB1*03:01, DRB1*04:01, DRB1*15:01, DRB3*01:01, DRB5*01:01, and DRB4*01:01 alleles, the recommended IEDB 2.22 prediction method was used. Henceforth, based on their ranking, the top-scored HTL and CTL epitopes that were found to be common for all of the selected corresponding HLA alleles were considered for further analyses. All the parameters were retained by opting for default during the T-cell epitope prediction. Later, B-cell epitopes of the proteins were predicted using the BepiPred linear epitope prediction method 2.0, maintaining all the default parameters and thus, the top-scored LBL epitopes containing more than ten amino acids were primarily regarded as potential candidates for further analysis^49^. Also, the conformational B-cell epitopes of the modeled 3D structure of the vaccine were predicted using IEDB ElliPro, an online server (http://tools.iedb.org/ellipro/) using the default parameters of a minimum score of 0.5 and a maximum distance of 6 angstroms^50^.

### Assessment of antigenicity, allergenicity, toxicity, and topology prediction of the epitopes

In this step, several methods for predicting their conservancy, antigenicity, allergenicity, and toxicity were used to evaluate the initially predicted T-cell and B-cell epitopes. To assess the conservancy of the chosen epitopes, the conservancy prediction method of the IEDB server (https://www.iedb.org/conservancy/) was used^51^. Also, the antigenicity determination tool VaxiJen v2.0 was used again for determination of antigenicity of the epitopes. Two different tools were then used, i.e. AllerTOP v2.0 (https://www.ddg-pharmfac.net/AllerTOP/) and AllergenFP v1.0 (http://ddg-pharmfac.net/AllergenFP/) to obtain the highest precision for prediction of allergenicity. Both of the tools are based on auto cross-covariance (ACC) transformation of protein sequences into uniform equal-length vectors. However, the AllerTOP v2.0 server has a better 88.7% prediction accuracy than the AllergenFP v1.0 server (87.9%)^52,53^. In addition, the ToxinPred (http://crdd.osdd.net/raghava/toxinpred/) server was used to predict toxicity for all epitopes by using the Support Vector Machine (SVM) prediction method to keep all the default parameters. Finally, using the TMHMM v2.0 server (https://services.healthtech.dtu.dk/service.php?TMHMM-2.0), the transmembrane topology prediction of all the epitopes was performed to predict whether the epitopes were exposed inside or outside, keeping the parameters at their default values. TMHMM uses an algorithm called N-best (or 1-best in this case) to predict the most probable location and orientation of transmembrane helices in the sequence^54^.

### Cytokine inducing capacity prediction of the epitopes

The induction capacity of the predicted HTL epitopes for interferon-γ (IFN-γ) was determined using the IFNepitope (http://crdd.osdd.net/raghava/ifnepitope/) server^55^. Based on analyzing a dataset that includes IFN-γ inducing and non-inducing peptides, the server determines the probable IFN-γ inducing epitopes. To determine the IFN-γ inducing capacity, the Design module and the Hybrid (Motif + SVM) prediction approach were used. In addition, IL-4 and IL-10 inducing HTL epitope properties were determined using the servers IL4pred (https://webs.iiitd.edu.in/raghava/il4pred/index.php) and IL10pred (http://crdd.osdd.net/raghava/IL-10pred/)^56,57^. The SVM method was used on both servers, where the default threshold values were kept at 0.2 and − 0.3, respectively.

### Conservancy and human proteome homology prediction

The conservancy analysis of the specified epitopes was conducted using the IEDB server’s epitope conservancy analysis module^51^. Afterwards, the homology of the human proteome epitopes was determined by the BLAST (BlastP) protein module of the BLAST tool (https://blast.ncbi.nlm.nih.gov/Blast.cgi), where Homo sapiens (taxid: 9606) was used for comparison, keeping all other default parameters. An e-value cut-off of 0.05 was set and epitopes were selected as non-homologous pathogen peptides that showed no hits below the e-value inclusion threshold^58^. The epitopes found to be highly antigenic, non-allergenic, non-toxic, fully conserved, and non-homologous to the human proteome were finally considered as the most promising epitopes, and only these selected epitopes were used in the construction of the vaccine.

### Population coverage and cluster analyses of the epitopes and their MHC alleles

The IEDB server (http://tools.iedb.org/population/) was used for analyzing the population coverage of the most promising epitopes across several HLA alleles in various regions around the world^59^. Denominated MHC restriction of T cell responses and polymorphic HLA combinations were considered in the analysis. All the parameters were maintained at their default conditions during the study. Furthermore, to evaluate the relationship between the selected MHC alleles, cluster analysis of the MHC alleles was done using the online tool MHCcluster 2.0 https://services.healthtech.dtu.dk/service.php?MHCcluster-2.0)^60^. During the study, 50,000 peptides to be used were retained, 100 bootstrap measurements were retained, and both HLA supertype (MHC Class-I) and HLA-DR (MHC class-II) representatives were chosen.

### Vaccine construction and biophysical property analyses

The most promising antigenic epitopes were linked with each other to create a fusion peptide using an adjuvant and linkers. Human beta-defensin-3 (hBD-3) was used as an adjuvant sequence that was linked to the epitopes by EAAAK linkers. The epitopes were also appended to the pan HLA-DR epitope (PADRE) sequence. In the conjugation of the CTL, HTL, and LBL epitopes, the AAY, GPGPG, and KK linkers were used, respectively. Once the vaccine construct was prepared, antigenicity of the sequences was measured using VaxiJen v2.0 server, keeping the threshold value fixed at 0.4. The findings of the Vaxijen v2.0 server were further cross-checked by the ANTIGENpro module of the SCRATCH protein predictor (http://scratch.proteomics.ics.uci.edu/), holding all the default parameters^61^, to achieve better prediction precision. Three different online methods were used to accurately perform the allergenicity of the vaccine i.e. AlgPred (http://crdd.osdd.net/raghava/algpred/), AllerTop v2.0 and AllergenFP v1.0, to ensure optimum prediction precision. The biophysical properties of the vaccine were then analysed ProtParam. Thereafter, the solubility of vaccine constructs was also estimated alongside the biophysical property study by the SOLpro module of the SCRATCH protein predictor (http://scratch.proteomics.ics.uci.edu/) and further validated by the Protein-Sol server (https://protein-sol.manchester.ac.uk/)^62^. All the parameters of the servers were maintained at their default values during the solubility review.

### Secondary and tertiary structure prediction of the vaccine construct

The vaccine construct was subjected to secondary structure prediction following biophysical property analysis. For this, several online tools were used to preserve all the default parameters, i.e. PSIPRED (http://bioinf.cs.ucl.uk/psipred/) (using the PSIPRED 4.0 prediction method), GOR IV (https://npsa-prabi.ibcp.fr/cgi-bin/npsa_automat.pl?page=/NPSA/npsa_gor4.html), SOPMA (https://npsa-pbil.ibcp.fr/cgi-bin/npsa_automat.pl?page=/NPSA/npsa_sopma.html) and SIMPA96 (https://npsa-prabi.ibcp.fr/cgi-bin/npsa_automat.pl?page=/NPSA/npsa_simpa96.html). To predict the percentages or quantities of amino acids in α helix, β-sheet, and coil structure formations, these servers are considered to be reliable, quick, and effective^63–67^. Moreover, 3D modelling of the vaccine construct was carried out using the RaptorX online server (https://bio.tools/raptorx). Using an easy and powerful template-based method^68^, the server predicts the tertiary structure of a query protein. RaptorX uses a deep learning method to enable distance-based protein folding^69^.

### Refinement and validation of tertiary structure of the vaccine

GalaxyWEB server (http://galaxy.seoklab.org/) using the GalaxyRefine module was used to refine the predicted model of the vaccine improving its resolution so that it resembles the native protein structure. The server uses dynamic simulation and the refinement approach is tested by CASP10 to refine the tertiary protein structures^70,71^. Furthermore, validation of the refined protein was carried out by analyzing the Ramachandran plot created by the PROCHECK (https://www.ebi.ac.uk/thornton-srv/software/PROCHECK/) tool^72^. Along with PROCHECK for protein validation, another online platform, ProSA-web (https://prosa.services.came.sbg.ac.at/prosa.php) was also used^73^. A z-score that expresses the consistency of a query protein structure is created by the PROCHECK server.

### Vaccine protein disulfide engineering analysis

In this experiment, the Disulfide by Design (DbD) 2 v12.2 (http://cptweb.cpt.wayne.edu/DbD2/) online tool was used to predict the locations and further design the disulfide bonds within the vaccine proteins^74^. The tool was developed using computational approaches to predict the protein structure, and the algorithm of this server accurately estimates the χ^3^ torsion angle based on 5the Cβ–Cβ distance using a geometric model derived from native disulfide bonds. The Caf-Cβ-Sγ angle is allowed some tolerance in the DbD2 server based on the wide range found in native disulfides. To facilitate the ranking process, DbD2 estimates an energy value for each potential disulfide and mutant PDB files may be generated for selected disulfides^75,76^. The χ^3^ angle was held at − 87° or + 97° ± 10 during the experiment to cast off various putative disulfides that were generated using the default angles of + 97° ± 30° and − 87° ± 30°. Additionally, the angle of Caf-Cβ-Sγ was set to its default value of 114.6° ± 10. Finally, to allow disulfide bridge formation, residue pairs with energy less than 2.2 kcal/mol were selected and mutated to cysteine residue^77^.

### Post-translational modification analysis

For posttranslational modification analysis of the vaccine construct comprising of the B-cell and T-cell epitopes, the NetNGlyc-1.0 (http://www.cbs.dtu.dk/services/NetNGlyc-1.0), NetOGlyc4.0 (http://www.cbs.dtu.dk/services/NetOGlyc-4.0), and NetPhos-3.1 (http://www.cbs.dtu.dk/services/NetPhos-3.1) servers were utilized. The NetNglyc server uses artificial neural networks to predict N-glycosylation sites in human proteins by examining the sequence context of Asn-Xaa-Ser/Thr sequons^78^. The NetOglyc server predicts mucin type GalNAc O-glycosylation sites in mammalian proteins using neural networks^79^. This server provides a list of probable glycosylation sites for each input sequence, together with their positions in the sequence and prediction confidence scores. Using ensembles of neural networks, the NetPhos 3.1 server predicts serine, threonine, or tyrosine phosphorylation sites in eukaryotic proteins^80^.

### Analysis of protein–protein docking

The vaccine protein was docked against several toll-like receptors (TLRs) in protein–protein docking analysis. In this study, different TLRs have been docked with the vaccine protein, i.e. TLR-1 (PDB ID: 6NIH), TLR-2 (PDB ID: 3A7C), TLR-3 (PDB ID: 2A0Z), TLR-4 (PDB ID: 4G8A), and TLR9 (PDB ID: 3WPF). ClusPro v2.0 (https://cluspro.bu.edu/login.php) was used to conduct the docking, where the lower energy score corresponds to the stronger binding affinity. Based on the following equation, the ClusPro server calculates the energy score:

$$E= 0.40Erep+ (-0.40Eatt) + 600Eelec +1.00EDARS$$

The repulsions and attraction energies owing to van der Waals interactions are denoted by E_rep_ and E_attr_, respectively, whereas E_elec_ signifies the electrostatic energy component. The Decoys' pairwise structure-based potential is represented by E_DARS_ as the Reference State (DARS) method^81,82^. Furthermore, another round of docking was carried out using the ZDOCK server (https://zdock.umassmed.edu/) which is a rigid-body protein–protein docking tool that employs a combination of shape complementarity, electrostatics, and statistical potential terms for scoring and uses the Fast Fourier Transform algorithm to enable an efficient global docking search on a 3D grid. In the most current benchmark version (Accelerating protein docking in ZDOCK utilizing an advanced 3D convolution library), ZDOCK achieves high predictive accuracy on protein–protein docking benchmarks, with > 70% success in the top 1000 predictions for rigid-body instances^83^.

### Molecular dynamics simulation studies and MM-PBSA calculations

The docked complexes from the ZDOCK server were used in MD simulations. The complexes being protein–protein in nature with multiple chains, the MD simulations were computationally expensive and performed on the HPC cluster at Bioinformatics Resources and Applications Facility (BRAF), C-DAC, Pune with Gromacs 2020.4^84^ MD simulation package. The CHARMM-36 force field parameters^85,86^ were employed to prepare the topology of protein chains. The system of each TLR along with the bound vaccine was solvated with the single point charge water model^87^ in the dodecahedron unit cells and neutralized with the addition of Na^+^ or Cl^−^ counter-ions. The solvated systems were initially energy minimized to relieve the steric clashes if any with the steepest descent criteria until the threshold (Fmax < 10 kJ/mol) was reached. These energy minimized systems were then equilibrated at constant volume and temperature conditions 300 K using modified Berendsen thermostat^88^ and then at constant volume and pressure Berendsen barostat^89^ for 100 ps each. The equilibrated systems were later subjected to 100 ns production phase MD simulations, where the modified Berendsen thermostat and Parrinello-Rahman barostat^90^ were used with covalent bonds restrained using the LINCS algorithm^91^. The long-range electrostatic interaction energies were measured with the cut-off of 12 Å, with the Particle Mesh Ewald method (PME)^92^. The resulting trajectories were analyzed for root mean square deviations (RMSD) in protein backbone atoms, root mean square fluctuations (RMSF) in the side chain atoms of individual chains in each protein complex, the radius of gyration (Rg), and several hydrogen bonds formed between vaccine protein chain and the respective TLR protein chain.

### Immune simulation studies

To forecast the immunogenicity and immune response profile of the proposed vaccine, an immune simulation analysis was performed. For the immune simulation study, the C-ImmSim server (http://150.146.2.1/CIMMSIM/index.php) was used to predict real-life immune interactions using machine learning techniques and PSSM (Position-Specific Scoring Matrix)^93^. During the experiment, all the variables except for the time steps were kept at their default parameters. However, the time steps at 1, 84, and 170 were retained (time step 1 is injection at time = 0), and the number of simulation steps was set to 1050. Thus, three injections at four-week intervals were administered to induce recurrent antigen exposure^94^.

### Codon adaptation and in silico cloning within *E. coli* system

Java Codon Adaptation Tool or JCat server (http://www.jcat.de/) was used for codon optimization^95^, and the optimized codon sequence was further analyzed for expression parameters, codon adaptation index (CAI), and GC-content %. The optimum CAI value is 1.0, while a score of > 0.8 is considered acceptable, and the optimum GC content ranges from 30 to 70%^96^. For in silico cloning simulation, the pETite vector plasmid was selected which contains a small ubiquitin-like modifier (SUMO) tag as well as a 6 × polyhistidine (6X-His)  tag^97^. The vaccine protein sequence was reverse-translated to the optimized DNA sequence by the server to which EaeI and StyI restriction sites were incorporated at the N-terminal and C-terminal sites, respectively. The newly adapted DNA sequence was then inserted between the EaeI and StyI restriction sites of the pETite vector using the SnapGene restriction cloning software (https://www.snapgene.com/free-trial/) to confirm the expression of the vaccine^98,99^.

### Prediction of the vaccine mRNA secondary structure

Two servers, i.e. Mfold (http://unafold.rna.albany.edu/?q=mfold) and RNAfold (http://rna.tbi.univie.ac.at/cgi-bin/RNAWebSuite/RNAfold.cgi), were used for the mRNA secondary structure prediction^100,101^. Both of these servers thermodynamically predict the mRNA secondary structures and provide each of the generated structures with minimum free energy ('G Kcal/mol'). To analyze the mRNA folding and secondary vaccine structure, the optimized DNA sequence was first taken from the JCat server and converted via the DNA <-> RNA-> Protein tool (http://biomodel.uah.es/en/lab/cybertory/analysis/trans.htm) to a possible RNA sequence. The RNA sequence was then gathered from the tool and utilized for prediction into the Mfold and RNAfold servers using the default settings for all the parameters.

### Supplementary Information

Supplementary Information.

## Data Availability

All the data generated during the experiment are provided in the manuscript/supplementary material.
