# Author Correction: A computational approach to design a polyvalent vaccine against human respiratory syncytial virus

**DOI:** 10.1038/s41598-024-66721-7

**Published:** 2024-07-08

**Authors:** Abu Tayab Moin, Md. Asad Ullah, Rajesh B. Patil, Nairita Ahsan Faruqui, Yusha Araf, Sowmen Das, Khaza Md. Kapil Uddin, Md. Shakhawat Hossain, Md. Faruque Miah, Mohammad Ali Moni, Dil Umme Salma Chowdhury, Saiful Islam

**Affiliations:** 1 Department of Genetic Engineering and Biotechnology, Faculty of Biological Sciences, University of Chittagong, Chattogram, Bangladesh; 2 Department of Biotechnology and Genetic Engineering, Faculty of Biological Sciences, Jahangirnagar University, Savar, Dhaka, Bangladesh; 3 Department of Pharmaceutical Chemistry, Sinhgad Technical Education Society’s, Sinhgad College of Pharmacy, Pune, Maharashtra India; 4 Biotechnology Program, Department of Mathematics and Natural Sciences, School of Data and Sciences, BRAC University, Dhaka, Bangladesh; 5 Department of Genetic Engineering and Biotechnology, School of Life Sciences, Shahjalal University of Science and Technology, Sylhet, Bangladesh; 6 Department of Computer Science and Engineering, School of Physical Sciences, Shahjalal University of Science and Technology, Sylhet, Bangladesh; 7 Bone Biology Division, The Garvan Institute of Medical Research, Darlinghurst, New South Wales, Australia; 8 WHO Collaborating Centre on eHealth, UNSW Digital Health, School of Public Health and Community Medicine, Faculty of Medicine, UNSW Sydney, Sydney, Australia; 9 Present Address: Artificial Intelligence and Data Science, Faculty of Health and Behavioural Sciences, School of Health and Rehabilitation Sciences, The University of Queensland, Brisbane, Australia; 10 Bangladesh Council of Scientific and Industrial Research (BCSIR), Chattogram Laboratories, Chattogram, Bangladesh

Correction to: *Scientific Reports* 10.1038/s41598-023-35309-y, published online 15 June 2023

The original version of this Article contained errors in the Acknowledgements section.

“With utmost gratitude, we extend our deepest appreciation to the distinguished couple, Alhajj Muhammad Abu Taher and Syeda Jasmin Akter, who have provided us with unwavering support, love, and encouragement throughout the research journey. We would also like to extend our heartfelt tribute to two remarkable individuals, Syeda Mumtaz Begum and the late Syeda Anwara Begum who had been a profound source of inspiration for us. Our utmost appreciation also goes to our esteemed supervisors and all the revered teachers at the Department of Genetic Engineering and Biotechnology, University of Chittagong, Chattogram, Bangladesh, for their constant guidance and mentorship. We are also immensely grateful to our collaborators and well-wishers from Bangladesh Council of Scientific and Industrial Research (BCSIR), Chattogram Laboratories, Chattogram, Bangladesh, Community of Biotechnology (COB), Dhaka, Bangladesh, International Foundation for Collaborative Research (IFCR), Chattogram, Bangladesh, and Chittagong University Research and Higher Study Society (CURHS), Chattogram, Bangladesh for their invaluable supports and contributions. Lastly, we dedicate this study to the young and ambitious student researchers of Bangladesh, who never falter in pursuing their dreams. To everyone who has extended their unwavering support and encouragement, we offer our heartfelt thanks.”

now reads:

“With utmost gratitude, we extend our deepest appreciation to the distinguished couple, Alhajj Muhammad Abu Taher and Syeda Jasmin Akter, who have provided us with unwavering support, love, and encouragement throughout the research journey. We would also like to extend our heartfelt tribute to two remarkable individuals, Syeda Mumtaz Begum and the late Syeda Anwara Begum, who had been a profound source of inspiration for us. Our utmost appreciation also goes to our esteemed supervisors and all the revered teachers at the Department of Genetic Engineering and Biotechnology, University of Chittagong, Chattogram, Bangladesh, for their constant guidance and mentorship. Lastly, we dedicate this study to the young and ambitious student researchers of Bangladesh, who never falter in pursuing their dreams. To everyone who has extended their unwavering support and encouragement, we offer our heartfelt thanks.”

The original Article has been corrected.

