# Supplementary material for: A computational approach to design a polyvalent vaccine against human respiratory syncytial virus
Source: Sci Rep. 2023 Jun 15;13:9702. doi: 10.1038/s41598-023-35309-y (PMC10272159; doi:10.1038/s41598-023-35309-y)
Supplement: Supplementary file 1 — Supplementary Information. [file 41598_2023_35309_MOESM1_ESM.docx]

**Supplementary Information**

**Supplementary Tables**

**S1 Table.** The antigenicity and physicochemical property analysis of the selected viral proteins. AN; antigenicity, pI; theoretical pI, II; instability index, AI; aliphatic index, GRAVY; grand average of hydropathicity.

| **Name of the Virus** | **Name of the Protein** | **UniProt accession ID the protein** | **AN**    **(threshold:**  **0.4)** | **pI** | **Number of positively charged amino acids** | **Number of negatively charged amino acids** | **Extinction coefficients**  **(in M^-1^ cm^-1^)** | **Estimated half-life**  **(in mammalian reticulocytes)** | **II** | **AI** | **GRAVY** |
| --- | --- | --- | --- | --- | --- | --- | --- | --- | --- | --- | --- |
| RSV-A | P protein | P03421 | Antigenic | 4.52 | 30 | 51 | 5960 | 30 h | 39.83 (stable) | 69.25 | -0.909 |
|  | N protein | P03418 | Antigenic | 6.87 | 45 | 46 | 29465 | 30 h | 37.58 (stable) | 94.30 | -0.212 |
|  | F protein | P03420 | Antigenic | 9.10 | 62 | 45 | 45810 | 30 h | 40.37 (unstable) | 99.97 | -0.028 |
|  | mG protein | P03423 | Antigenic | 9.90 | 35 | 15 | 15720 | 30 h | 40.64 (unstable) | 65.13 | -0.662 |
| RSV-B | P protein | O42062 | Antigenic | 4.52 | 31 | 52 | 5960 | 30 h | 42.93 (unstable) | 71.70 | -0.827 |
|  | N protein | O42053 | Antigenic | 6.53 | 45 | 47 | 30955 | 30 h | 36.84 (stable) | 95.78 | -0.218 |
|  | F protein | O36634 | Antigenic | 9.08 | 60 | 44 | 50155 | 30 h | 39.51 (stable) | 102.35 | -0.033 |
|  | mG protein | O36633 | Antigenic | 10.04 | 40 | 16 | 10220 | 30 h | 27.77 (stable) | 61.97 | -0.825 |

**S2 Table.** List of the predicted MHC class-I, MHC class-II, and B-cell epitopes and their topology, antigenicity, allergenicity, toxicity, conservancy, and human homology analyses of the epitopes of phosphoprotein (P protein). AN; antigenicity, AG; allergenicity, CN; conservancy, TT; transmembrane topology.

| **MHC class** | **Peptide** | **Start** | **End** | **AN** | **AG** | **CN** | **Homology to human proteome** | **Toxicity** | **IFN-gamma inducing ability** | **IL-4 inducing ability** | **IL-10 inducing ability** | **TT** |
| --- | --- | --- | --- | --- | --- | --- | --- | --- | --- | --- | --- | --- |
| **MHC class-I** | VSLNPTSEK | 214 | 222 | Antigen | Non-allergen | Conserved | Non-homolog | Non-toxic | NA | NA | NA | Outside |
|  | ASAGPTSAR | 155 | 163 | Antigen | Allergen | Conserved | Non-homolog | Non-toxic | NA | NA | NA | Inside |
|  | NSIDIEVTK | 44 | 52 | Antigen | Allergen | Conserved | Non-homolog | Non-toxic | NA | NA | NA | Inside |
|  | QTNDNITAR | 126 | 134 | Antigen | Non-allergen | Conserved | Non-homolog | Non-toxic | NA | NA | NA | Inside |
|  | GLREEMIEK | 172 | 180 | Non-antigen | Allergen | Conserved | Non-homolog | Non-toxic | NA | NA | NA | Outside |
|  | FTSPKDPKK | 28 | 36 | Non-antigen | Non-allergen | Conserved | Non-homolog | Non-toxic | NA | NA | NA | Inside |
|  | ATKFLESIK | 17 | 25 | Non-antigen | Allergen | Conserved | Non-homolog | Non-toxic | NA | NA | NA | Inside |
|  | AGNKPNYQR | 73 | 81 | Non-antigen | Allergen | Non-conserved | Non-homolog | Non-toxic | NA | NA | NA | Inside |
|  | RLRNEESEK | 197 | 205 | Non-antigen | Non-allergen | Conserved | Non-homolog | Non-toxic | NA | NA | NA | Inside |
|  | KFLESIKGK | 19 | 27 | Non-antigen | Non-allergen | Conserved | Non-homolog | Non-toxic | NA | NA | NA | Inside |
| **MHC class-II** | SEILGMLHTLVVASA | 143 | 157 | Non-antigen | Non-allergen | Conserved | Non-homolog | Non-toxic | Non-inducer | Non-inducer | Non-inducer | Outside |
|  | EILGMLHTLVVASAG | 144 | 158 | Non-antigen | Non-allergen | Conserved | Non-homolog | Non-toxic | Non-inducer | Non-inducer | Non-inducer | Outside |
|  | ILGMLHTLVVASAGP | 145 | 159 | Non-antigen | Non-allergen | Conserved | Non-homolog | Non-toxic | Non-inducer | Non-inducer | Non-inducer | Outside |
|  | EAMARLRNEESEKMA | 193 | 207 | Non-antigen | Allergen | Conserved | Non-homolog | Non-toxic | Non-inducer | Inducer | Inducer | Inside |
|  | LEAMARLRNEESEKM | 192 | 206 | Antigen | Allergen | Conserved | Non-homolog | Non-toxic | Non-inducer | Inducer | Inducer | Inside |
|  | LGMLHTLVVASAGPT | 146 | 160 | Antigen | Non-allergen | Conserved | Non-homolog | Non-toxic | Inducer | Inducer | Non-inducer | Inside |
|  | LSEILGMLHTLVVAS | 131 | 145 | Non-antigen | Allergen | Conserved | Non-homolog | Non-toxic | Non-inducer | Non-inducer | Non-inducer | Outside |
|  | LHTLVVASAGPTSAR | 149 | 163 | Antigen | Non-allergen | Conserved | Non-homolog | Non-toxic | Non-inducer | Inducer | Inducer | Inside |
|  | MARLRNEESEKMAKD | 195 | 209 | Non-antigen | Non-allergen | Conserved | Non-homolog | Non-toxic | Non-inducer | Inducer | Non-inducer | Inside |
|  | ARLRNEESEKMAKDT | 196 | 210 | Non-antigen | Non-allergen | Conserved | Non-homolog | Non-toxic | Inducer | Inducer | Non-inducer | Inside |
| **B-cell epitopes** | PEFHGEDANNR | 6 | 16 | Antigen | Non-allergen | Conserved | Non-homolog | Non-toxic | NA | NA | NA | Outside |
|  | GKFTSPKDPKKKDS | 26 | 39 | Non-antigen | Non-allergen | Conserved | Non-homolog | Non-toxic | NA | NA | NA | Outside |
|  | EVTKESPITSNSTIINPTNETDDTAGNKPNYQRK | 49 | 82 | Antigen | Non-allergen | Conserved | Non-homolog | Non-toxic | NA | NA | NA | Inside |
|  | SFKEDPTPSDNPFS | 86 | 99 | Antigen | Non-allergen | Conserved | Non-homolog | Non-toxic | NA | NA | NA | Outside |
|  | IETFDNNEEESSYSYEEINDQTNDNIT | 106 | 132 | Antigen | Non-allergen | Non-conserved | Non-homolog | Non-toxic | NA | NA | NA | Outside |
|  | SAGPTSARDGI | 156 | 166 | Antigen | Allergen | Conserved | Non-homolog | Non-toxic | NA | NA | NA | Inside |
|  | RNEESEKMAKDTSDEVSLNPTSEK | 200 | 222 | Antigen | Non-allergen | Conserved | Non-homolog | Non-toxic | NA | NA | NA | Inside |

**S3 Table.** List of the predicted MHC class-I, MHC class-II, and B-cell epitopes and their topology, antigenicity, allergenicity, toxicity, conservancy, and human homology analyses of the epitopes of nucleoprotein (N protein). AN; antigenicity, AG; allergenicity, CN; conservancy, TT; transmembrane topology.

| **MHC class** | **Peptide** | **Start** | **End** | **AN** | **AG** | **CN** | **Homology to human proteome** | **Toxicity** | **IFN-gamma inducing ability** | **IL-4 inducing ability** | **IL-10 inducing ability** | **TT** |
| --- | --- | --- | --- | --- | --- | --- | --- | --- | --- | --- | --- | --- |
| **MHC class-I** | NSFYEVFEK | 207 | 2015 | Non-antigen | Allergen | Conserved | Non-homolog | Non-toxic | NA | NA | NA | Outside |
|  | SSSKYTIQR | 19 | 27 | Non-antigen | Non-allergen | Conserved | Non-homolog | Non-toxic | NA | NA | NA | Inside |
|  | CIAALVITK | 162 | 170 | Antigen | Non-allergen | Conserved | Non-homolog | Non-toxic | NA | NA | NA | Inside |
|  | MLRWGVLAK | 257 | 265 | Antigen | Allergen | Conserved | Non-homolog | Non-toxic | NA | NA | NA | Outside |
|  | AIKHQLNPK | 377 | 385 | Antigen | Allergen | Conserved | Non-homolog | Non-toxic | NA | NA | NA | Inside |
|  | FTGLIGMLY | 61 | 69 | Non-antigen | Non-allergen | Conserved | Non-homolog | Non-toxic | NA | NA | NA | Outside |
|  | RSGLTAVIR | 176 | 184 | Antigen | Non-allergen | Conserved | Non-homolog | Non-toxic | NA | NA | NA | Inside |
|  | SGLTAVIRR | 177 | 185 | Non-antigen | Allergen | Conserved | Non-homolog | Non-toxic | NA | NA | NA | Inside |
|  | LITEDANHK | 52 | 60 | Non-antigen | Allergen | Conserved | Non-homolog | Non-toxic | NA | NA | NA | Inside |
|  | SVKNIMLGH | 266 | 274 | Antigen | Non-allergen | Conserved | Non-homolog | Non-toxic | NA | NA | NA | Inside |
| **MHC class-II** | AGFYHILNNPKASLL | 298 | 312 | Non-antigen | Non-allergen | Conserved | Non-homolog | Non-toxic | Non-inducer | Non-inducer | Non-induce | Outside |
|  | EAGFYHILNNPKASL | 297 | 311 | Non-antigen | Non-allergen | Conserved | Non-homolog | Non-toxic | Non-inducer | Inducer | Non-induce | Outside |
|  | GEAGFYHILNNPKAS | 296 | 310 | Non-antigen | Non-allergen | Conserved | Non-homolog | Non-toxic | Non-inducer | Inducer | Non-induce | Outside |
|  | GGEAGFYHILNNPKA | 295 | 309 | Non-antigen | Non-allergen | Conserved | Non-homolog | Non-toxic | Non-inducer | Non-inducer | Non-induce | Outside |
|  | GFYHILNNPKASLLS | 299 | 313 | Non-antigen | Allergen | Conserved | Non-homolog | Non-toxic | Non-inducer | Non-inducer | Non-induce | Outside |
|  | EVLTLASLTTEIQIN | 112 | 126 | Antigen | Non-allergen | Conserved | Non-homolog | Non-toxic | Non-inducer | Inducer | Non-inducer | Outside |
|  | FEVLTLASLTTEIQI | 111 | 125 | Antigen | Allergen | Conserved | Non-homolog | Non-toxic | Non-inducer | Inducer | Non-inducer | Outside |
|  | LGGEAGFYHILNNPK | 294 | 308 | Non-antigen | Non-allergen | Conserved | Non-homolog | Non-toxic | Non-inducer | Inducer | Non-inducer | Outside |
|  | VLTLASLTTEIQINI | 113 | 127 | Antigen | Allergen | Conserved | Non-homolog | Non-toxic | Non-inducer | Non-inducer | Non-inducer | Outside |
|  | LTLASLTTEIQINIE | 114 | 128 | Antigen | Allergen | Conserved | Non-homolog | Non-toxic | Non-inducer | Inducer | Non-inducer | Outside |
| **B-cell epitopes** | YTIQRSTGDSIDTPNYDVQ | 23 | 40 | Non-antigen | Allergen | Conserved | Non-homolog | Non-toxic | NA | NA | NA | Inside |
|  | YHVKANGVDVTTHRQDING | 88 | 106 | Antigen | Allergen | Conserved | Non-homolog | Non-toxic | NA | NA | NA | Inside |
|  | EVAPEYRHDSPD | 144 | 155 | Antigen | Non-allergen | Conserved | Non-homolog | Non-toxic | NA | NA | NA | Outside |
|  | EYRGTPRNQDLYDA | 336 | 349 | Antigen | Non-allergen | Conserved | Non-homolog | Non-toxic | NA | NA | NA | Inside |

**S4 Table.** List of the predicted MHC class-I, MHC class-II, and B-cell epitopes and their topology, antigenicity, allergenicity, toxicity, conservancy, and human homology analyses of the epitopes of fusion glycoprotein (F protein). AN; antigenicity, AG; allergenicity, CN; conservancy, TT; transmembrane topology.

| **MHC class** | **Peptide** | **Start** | **End** | **AN** | **AG** | **CN** | **Homology to human proteome** | **Toxicity** | **IFN-gamma inducing ability** | **IL-4 inducing ability** | **IL-10 inducing ability** | **TT** |
| --- | --- | --- | --- | --- | --- | --- | --- | --- | --- | --- | --- | --- |
| **MHC class-I** | ASISQVNEK | 490 | 498 | Antigen | Allergen | Conserved | Non-homolog | Non-toxic | NA | NA | NA | Inside |
|  | RSTPVTLSK | 553 | 561 | Antigen | Allergen | Conserved | Non-homolog | Non-toxic | NA | NA | NA | Inside |
|  | KTNVTLSKK | 124 | 132 | Antigen | Non-allergen | Conserved | Non-homolog | Non-toxic | NA | NA | NA | Inside |
|  | KSALLSTNK | 168 | 176 | Antigen | Non-allergen | Conserved | Non-homolog | Non-toxic | NA | NA | NA | Inside |
|  | IASGVAVSK | 148 | 156 | Antigen | Non-allergen | Conserved | Non-homolog | Non-toxic | NA | NA | NA | Outside |
|  | KQLLPIVNK | 201 | 209 | Antigen | Non-allergen | Conserved | Non-homolog | Non-toxic | NA | NA | NA | Inside |
|  | ITIELSNIK | 57 | 65 | Antigen | Non-allergen | Conserved | Non-homolog | Non-toxic | NA | NA | NA | Inside |
|  | GVIDTPCWK | 307 | 315 | Non-antigen | Non-allergen | Conserved | Non-homolog | Non-toxic | NA | NA | NA | Inside |
|  | VSVGNTLYY | 450 | 458 | Antigen | Allergen | Conserved | Non-homolog | Non-toxic | NA | NA | NA | Outside |
|  | LTSKVLDLK | 188 | 196 | Antigen | Non-allergen | Conserved | Non-homolog | Non-toxic | NA | NA | NA | Outside |
| **MHC class-II** | IVIIVILLSLIAVGL | 532 | 546 | Antigen | Non-allergen | Conserved | Non-homolog | Non-toxic | Non-inducer | Non-inducer | Inducer | Outside |
|  | VIIVILLSLIAVGLL | 533 | 547 | Antigen | Non-allergen | Conserved | Non-homolog | Non-toxic | Non-inducer | Non-inducer | Inducer | Outside |
|  | IIIVIIVILLSLIAV | 531 | 545 | Antigen | Non-allergen | Conserved | Non-homolog | Non-toxic | Non-inducer | Non-inducer | Non-inducer | Outside |
|  | QELDKYKNAVTELQL | 81 | 95 | Non-antigen | Non-antigen | Conserved | Non-homolog | Non-toxic | Inducer | Inducer | Non-inducer | Inside |
|  | LDKYKNAVTELQLLM | 83 | 97 | Non-antigen | Non-antigen | Conserved | Non-homolog | Non-toxic | Inducer | Non-inducer | Non-inducer | Inside |
|  | DKYKNAVTELQLLMQ | 84 | 98 | Non-antigen | Non-antigen | Conserved | Non-homolog | Non-toxic | Inducer | Inducer | Non-inducer | Inside |
|  | ELDKYKNAVTELQLL | 82 | 96 | Non-antigen | Non-antigen | Conserved | Non-homolog | Non-toxic | Inducer | Non-inducer | Non-inducer | Inside |
|  | KQELDKYKNAVTELQ | 80 | 94 | Non-antigen | Non-antigen | Conserved | Non-homolog | Non-toxic | Inducer | Inducer | Non-inducer | Inside |
|  | TIIIVIIVILLSLIA | 529 | 543 | Antigen | Non-allergen | Conserved | Non-homolog | Non-toxic | Non-inducer | Non-inducer | Non-inducer | Inside |
|  | EEFYQSTCSAVSKGY | 30 | 44 | Antigen | Non-allergen | Conserved | Non-homolog | Non-toxic | Inducer | Inducer | Non-inducer | Inside |
| **B-cell epitopes** | STPPTNNRARR | 99 | 109 | Non-antigen | Non-allergen | Conserved | Non-homolog | Non-toxic | NA | NA | NA | Inside |
|  | KTKCTASNKNR | 419 | 429 | Antigen | Allergen | Conserved | Non-homolog | Non-toxic | NA | NA | NA | Inside |
|  | DYVSNKGMDTVSV | 440 | 452 | Antigen | Allergen | Conserved | Non-homolog | Non-toxic | NA | NA | NA | Inside |
|  | VFPSDEFDASISQVNEK | 482 | 498 | Antigen | Non-allergen | Conserved | Non-homolog | Non-toxic | NA | NA | NA | Outside |

**S5 Table.** List of the predicted MHC class-I, MHC class-II, and B-cell epitopes and their topology, antigenicity, allergenicity, toxicity, conservancy, and human homology analyses of the epitopes of major surface glycoprotein (mG protein). AN; antigenicity, AG; allergenicity, CN; conservancy, TT; transmembrane topology.

| **MHC class** | **Peptide** | **Start** | **End** | **AN** | **AG** | **CN** | **Homology to human proteome** | **Toxicity** | **IFN-gamma inducing ability** | **IL-4 inducing ability** | **IL-10 inducing ability** | **TT** |
| --- | --- | --- | --- | --- | --- | --- | --- | --- | --- | --- | --- | --- |
| **MHC class-I epitopes** | STLQSTTVK | 124 | 132 | Antigen | Allergen | Conserved | Non-homolog | Non-toxic | NA | NA | NA | Inside |
|  | KSKEVPTTK | 221 | 229 | Antigen | Allergen | Conserved | Non-homolog | Non-toxic | NA | NA | NA | Inside |
|  | KTTTKPTKK | 197 | 205 | Antigen | Allergen | Conserved | Non-homolog | Non-toxic | NA | NA | NA | Inside |
|  | AICKRIPNK | 184 | 192 | Non-antigen | Non-allergen | Conserved | Non-homolog | Non-toxic | NA | NA | NA | Inside |
|  | TTTQTQPSK | 137 | 145 | Antigen | Non-allergen | Conserved | Non-homolog | Non-toxic | NA | NA | NA | Inside |
|  | RIPNKKPGK | 188 | 196 | Non-allergen | Allergen | Conserved | Non-homolog | Non-toxic | NA | NA | NA | Inside |
|  | IQDATSQIK | 76 | 84 | Non-antigen | Non-allergen | Conserved | Non-homolog | Non-toxic | NA | NA | NA | Inside |
|  | IFIASANHK | 60 | 68 | Antigen | Non-allergen | Conserved | Non-homolog | Non-toxic | NA | NA | NA | Inside |
|  | QIKNTTPTY | 82 | 90 | Antigen | Allergen | Conserved | Non-homolog | Non-toxic | NA | NA | NA | Inside |
|  | SQVSTTSEY | 277 | 285 | Antigen | Non-allergen | Conserved | Non-homolog | Non-toxic | NA | NA | NA | Inside |
| **MHC class-II epitopes** | ILAMIISTSLIIAAI | 45 | 59 | Non-antigen | Non-allergen | Conserved | Non-homolog | Non-toxic | Non-inducer | Non-inducer | Inducer | Outside |
|  | LSILAMIISTSLIIA | 43 | 57 | Antigen | Non-allergen | Conserved | Non-homolog | Non-toxic | Non-inducer | Inducer | Non-inducer | Outside |
|  | SILAMIISTSLIIAA | 44 | 58 | Non-antigen | Non-allergen | Conserved | Non-homolog | Non-toxic | Non-inducer | Inducer | Non-inducer | Outside |
|  | TLSILAMIISTSLII | 42 | 56 | Antigen | Non-allergen | Conserved | Non-homolog | Non-toxic | Non-inducer | Inducer | Non-inducer | Outside |
|  | IAAIIFIASANHKVT | 56 | 70 | Antigen | Non-allergen | Conserved | Non-homolog | Non-toxic | Non-inducer | Non-inducer | Non-inducer | Outside |
|  | QNPQLGISPSNPSEI | 93 | 107 | Antigen | Non-allergen | Conserved | Non-homolog | Non-toxic | Non-inducer | Inducer | Non-inducer | Outside |
|  | AAIIFIASANHKVTP | 57 | 71 | Antigen | Non-allergen | Conserved | Non-homolog | Non-toxic | Non-inducer | Non-inducer | Non-inducer | Outside |
|  | SQITTILASTTPGVK | 109 | 123 | Antigen | Allergen | Conserved | Non-homolog | Non-toxic | Non-inducer | Non-inducer | Non-inducer | Inside |
|  | ITTILASTTPGVKST | 111 | 125 | Antigen | Allergen | Conserved | Non-homolog | Non-toxic | Non-inducer | Inducer | Non-inducer | Inside |
|  | QITTILASTTPGVKS | 110 | 124 | Antigen | Allergen | Conserved | Non-homolog | Non-toxic | Non-inducer | Inducer | Non-inducer | Inside |
| **B-cell epitopes** | MSKNKDQRTAKTLER | 1 | 15 | Non-antigen | Allergen | Conserved | Non-homolog | Non-toxic | NA | NA | NA | Inside |
|  | HKVTPTTAIIQD | 67 | 78 | Non-antigen | Non-allergen | Conserved | Non-homolog | Non-toxic | NA | NA | NA | Inside |
|  | TSQIKNTTPTYLTQNPQLGISPSNPSEITS | 80 | 109 | Antigen | Non-allergen | Conserved | Non-homolog | Non-toxic | NA | NA | NA | Inside |
|  | STTPGVKSTLQSTTVKTKNTTTTQTQPSKPTTKQRQNKPPSKPNND | 117 | 162 | Antigen | Allergen | Conserved | Non-homolog | Non-toxic | NA | NA | NA | Inside |
|  | IPNKKPGKKTTTKPTKKPTLKTTKKDPKPQTTKSKEVPTTKPTEEPTINTT | 189 | 239 | Antigen | Non-allergen | Conserved | Non-homolog | Non-toxic | NA | NA | NA | Outside |
|  | SNTTGNPELTSQ | 250 | 261 | Antigen | Non-allergen | Conserved | Non-homolog | Non-toxic | NA | NA | NA | Inside |

**S6 Table.** The list of the predicted conformational B-cell epitopes of the vaccine, with their scores.

| **No** | **Residues** | **No of residues** | **Score** |
| --- | --- | --- | --- |
| **01** | A:E1, A:A3, A:G6, A:I7, A:N9, A:T10, A:L11, A:Q12, A:K13, A:Y14, A:Y15, A:C16, A:V18, A:R19, A:G20, A:G21, A:R22, A:E32, A:Q34, A:I35, A:E51, A:A52, A:A54, A:K55, A:A56, A:F58, A:V59, A:A60, A:A61, A:W62, A:T63, A:K65, A:A66, A:A67, A:A68, A:A69, A:A70, A:Y71, A:V72, A:S73, A:L74, A:N75, A:P76, A:T77, A:S78, A:E79, A:K80, A:A81, A:Y83, A:Q84, A:T85, A:I115, A:R116, A:A117, A:A118, A:Y119, A:H128, A:Y143, A:K144, A:S145, A:A146, A:L147, A:N151, A:K152, A:A153, A:A154, A:Y155, A:I156, A:A157, A:S158, A:G159, A:V160, A:A161, A:V162, A:S163, A:K164, A:A165, A:A166, A:Y167, A:K168, A:Q169, A:L170, A:L171, A:P172, A:I173, A:V174, A:N175, A:K176, A:A177, A:A178, A:Y179, A:I180, A:T181, A:I182, A:E183, A:L184, A:S185, A:N186, A:I187, A:K188, A:A189, A:A190, A:T206, A:Q207, A:T208, A:Q209, A:P210, A:S211, A:K212, A:A213, A:A214, A:I216, A:F217, A:A219, A:S220, A:A221, A:N222, A:H223, A:K224, A:G225, A:P226, A:G227, A:P228, A:G229, A:M232, A:L233, A:H234, A:V237, A:V238, A:A239, A:S240, A:A241, A:G242, A:P243, A:T244, A:G245, A:A256, A:S257, A:A258, A:G259, A:P260, A:T261, A:S262, A:A263, A:R264, A:G265, A:P266, A:G267, A:P268, A:G269, A:E270, A:V271, A:L272, A:T273, A:L274, A:A275, A:S276, A:L277, A:T278, A:T279, A:E280, A:I281, A:Q282, A:I283, A:N284, A:G285, A:P286, A:G287, A:P288, A:G289, A:I290, A:V291, A:I292, A:I293, A:G305, A:P306, A:G307, A:P308, A:G309, A:V310, A:I311, A:I312, A:V313, A:I314, A:L315, A:L316, A:S317, A:L318, A:I319, A:A320, A:V321, A:G322, A:F332, A:Y333, A:Q334, A:S335, A:T336, A:C337, A:S338, A:A339, A:V340, A:S341, A:K342, A:G343, A:Y344, A:G345, A:P346, A:G347, A:P348, A:G349, A:L350, A:I352, A:L353, A:L371, A:S372, A:P386, A:G387, A:P388, A:G389, A:Q390, A:N391, A:P392, A:Q393, A:K419, A:E420, A:V421, A:T422, A:E424, A:S425, A:P426, A:T437, A:N438, A:E439, A:T440, A:Y450, A:Q451, A:R452, A:K453, A:K454, A:K455, A:S456, A:F457, A:K458, A:E459, A:D460, A:P461, A:T462, A:P463, A:S464, A:D465, A:N466, A:P467, A:F468, A:S469, A:K470, A:K471, A:R472, A:N473, A:E474, A:E475, A:S476, A:E477, A:K478, A:M479, A:A480, A:K481, A:D482, A:T483, A:S484, A:D485, A:E486, A:V487, A:S488, A:L489, A:N490, A:P491, A:T492, A:S493, A:E494, A:K495, A:K496, A:E498, A:V499, A:A500, A:P501, A:E502, A:Y503, A:H505, A:D506, A:S507, A:P508, A:D509, A:K510, A:K511, A:E512, A:Y513, A:R514, A:G515, A:T516, A:P517, A:R518, A:N519, A:Q520, A:D521, A:L522, A:Y523, A:D524, A:A525, A:K526, A:K527, A:V528, A:F529, A:P530, A:S531, A:D532, A:E533, A:F534, A:D535, A:S548, A:E573, A:G585, A:K586, A:T589, A:T590, A:K591, A:T593, A:K594, A:K595, A:P596, A:T597, A:L598, A:K599, A:T600, A:T601, A:K602, A:K603, A:D604, A:P605, A:K606, A:P607, A:Q608, A:T609, A:T610, A:K611, A:S612, A:K613, A:E614, A:V615, A:P616, A:T617, A:T618, A:K619, A:P620, A:T621, A:E622, A:E623, A:P624, A:T625, A:I626, A:N627, A:T628, A:T629, A:K630, A:K631, A:S632, A:N633, A:T634, A:T635, A:G636, A:N637, A:P638, A:E639, A:L640, A:T641, A:S642, A:Q643, A:K644, A:K645, A:A646, A:K647, A:F648, A:V649, A:A650, A:A651, A:W652, A:T653, A:L654, A:K655, A:A656, A:A657, A:A658, A:A659, A:A660, A:Y661 | 394 | 0.675 |
| **02** | A:R41, A:R43, A:K44, A:R47 | 4 | 0.514 |
| **03** | A:N561, A:P562, A:Q563, A:L564, A:G565, A:I566, A:S567, A:P568, A:S569, A:I574, A:S576, A:K577, A:K578 | 13 | 0.506 |

**S7 Table.** Data of Hydrogen bonds formed at the interface of TLRs and vaccine chain

| **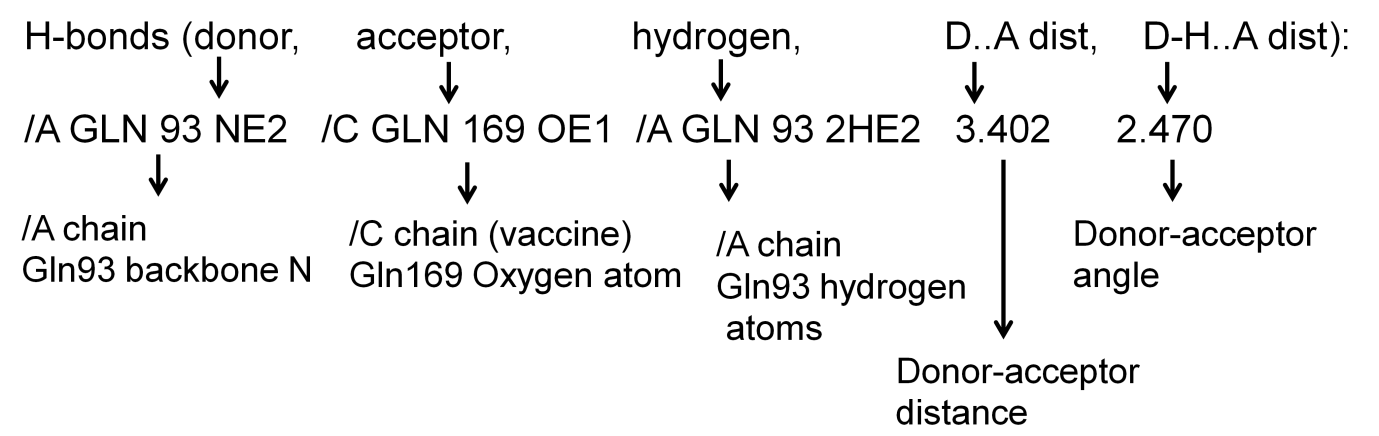** |
| --- |
| #####################################################################  TLR1_0.pdb (Equilibrated snapshot of TLR1/chainA & Vaccine chainC)  8 H-bonds  H-bonds (donor, acceptor, hydrogen, D..A dist, D-H..A dist):  /A GLN 93 NE2 /C GLN 169 OE1 /A GLN 93 2HE2 3.402 2.470  /A LYS 117 NZ /C GLU 270 OE1 /A LYS 117 HZ3 3.421 2.692  /A LYS 117 NZ /C GLU 270 OE2 /A LYS 117 HZ3 2.898 1.887  /A GLN 140 NE2 /C VAL 271 O /A GLN 140 2HE2 2.668 1.793  /C TYR 167 OH /A LYS 90 O /C TYR 167 HH 2.704 1.766  /C LYS 176 NZ /A GLN 93 O /C LYS 176 HZ1 2.681 1.792  /C ALA 178 N /A GLU 94 OE1 /C ALA 178 HN 2.959 2.027  /C THR 273 N /A GLN 140 OE1 /C THR 273 HN 3.011 2.114  #######################################################################  TLR1_last.pdb (Last snapshot of TLR1/chainA & Vaccine chain C)  3 H-bonds  H-bonds (donor, acceptor, hydrogen, D..A dist, D-H..A dist):  /C GLN 169 NE2 /A LYS 90 O /C GLN 169 2HE2 2.970 2.020  /C GLN 169 NE2 /A GLN 93 OE1 /C GLN 169 1HE2 3.005 2.054  /C LYS 176 NZ /A GLU 94 OE1 /C LYS 176 HZ2 2.797 1.762  ########################################################################  TLR1_0.pdb (Equilibrated snapshot of TLR1/chainB & Vaccine chain C)  12 H-bonds  H-bonds (donor, acceptor, hydrogen, D..A dist, D-H..A dist):  /B LYS 300 NZ /C GLU 270 OE2 /B LYS 300 HZ1 3.037 2.081  /B ASN 327 ND2 /C GLY 265 O /B ASN 327 1HD2 3.279 2.625  /B LYS 346 NZ /C TYR 179 O /B LYS 346 HZ1 2.797 1.762  /B LYS 346 NZ /C PRO 266 O /B LYS 346 HZ2 2.764 1.780  /B GLY 369 N /C GLU 183 OE2 /B GLY 369 HN 3.293 2.574  /B GLN 396 NE2 /C SER 185 O /B GLN 396 1HE2 3.462 2.881  /B GLN 396 NE2 /C ASN 186 OD1 /B GLN 396 2HE2 3.693 2.765  /C TYR 179 OH /B THR 372 OG1 /C TYR 179 HH 2.868 1.935  /C ARG 264 NE /B SER 295 OG /C ARG 264 HE 2.978 2.017  /C ARG 264 NH1 /B ASP 293 OD1 /C ARG 264 2HH1 3.268 2.588  /C ARG 264 NH2 /B ASP 293 OD1 /C ARG 264 2HH2 2.866 1.881  /C LYS 453 NZ /B ASP 291 OD2 /C LYS 453 HZ3 2.647 1.614  ##########################################################################  TLR1_last.pdb (Last snapshot of TLR1/chainB & Vaccine chain C)  3 H-bonds  H-bonds (donor, acceptor, hydrogen, D..A dist, D-H..A dist):  /B SER 298 OG /C LEU 272 O /B SER 298 HG1 3.001 2.135  /B ASN 327 ND2 /C GLY 269 O /B ASN 327 1HD2 3.441 2.626  /C LEU 272 N /B THR 297 O /C LEU 272 HN 2.734 1.801 |
| ###########################################################################  TLR2_0.pdb (Equilibrated snapshot of TLR2/chainA & Vaccine chain B)  4 H-bonds  H-bonds (donor, acceptor, hydrogen, D..A dist, D-H..A dist):  /A TYR 332 OH /B ALA 178 O /A TYR 332 HH 2.861 2.131  /A PHE 349 N /B GLU 270 O /A PHE 349 HN 3.531 2.571  /B LYS 176 NZ /A PHE 322 O /B LYS 176 HZ3 2.575 1.584  /B LYS 176 NZ /A LEU 324 O /B LYS 176 HZ2 2.731 1.730  ###########################################################################  TLR2_last.pdb (Last snapshot of TLR2/chainA & Vaccine chain B)  4 H-bonds  H-bonds (donor, acceptor, hydrogen, D..A dist, D-H..A dist):  /B LYS 152 NZ /A ASP 294 OD2 /B LYS 152 HZ2 2.729 1.785  /B ALA 177 N /A LEU 324 O /B ALA 177 HN 2.802 1.969  /B TYR 179 N /A TYR 326 O /B TYR 179 HN 2.999 2.041  /B LYS 188 NZ /A ASP 327 OD1 /B LYS 188 HZ1 2.753 1.772 |
| ###########################################################################  TLR3_0.pdb (Equilibrated snapshot of TLR3/chainA & Vaccine chain B)  5 H-bonds  H-bonds (donor, acceptor, hydrogen, D..A dist, D-H..A dist):  /A GLN 299 NE2 /B SER 469 O /A GLN 299 2HE2 2.873 1.880  /A HIS 406 NE2 /B PHE 58 O /A HIS 406 HE2 3.253 2.348  /B LYS 5 NZ /A ASP 292 OD2 /B LYS 5 HZ1 3.197 2.261  /B ARG 41 NH2 /A ASN 291 OD1 /B ARG 41 1HH2 3.344 2.509  /B ARG 41 NH2 /A SER 315 OG /B ARG 41 2HH2 2.838 1.856  ###########################################################################  TLR3_last.pdb (Last snapshot of TLR3/chainA & Vaccine chain B)  11 H-bonds  H-bonds (donor, acceptor, hydrogen, D..A dist, D-H..A dist):  /A ASN 247 ND2 /B THR 516 O /A ASN 247 2HD2 3.350 2.762  /A ASN 275 ND2 /B LYS 471 O /A ASN 275 2HD2 2.771 1.878  /A GLN 299 NE2 /B SER 469 O /A GLN 299 2HE2 3.012 2.057  /B LYS 5 NZ /A ASP 292 OD1 /B LYS 5 HZ3 2.708 1.669  /B SER 469 OG /A GLN 299 OE1 /B SER 469 HG1 2.581 1.661  /B LYS 471 N /A GLN 299 OE1 /B LYS 471 HN 3.207 2.235  /B LYS 471 NZ /A LYS 272 O /B LYS 471 HZ1 3.120 2.452  /B LYS 471 NZ /A ASN 275 OD1 /B LYS 471 HZ2 2.741 1.720  /B ARG 518 NH1 /A GLU 244 OE1 /B ARG 518 2HH1 2.756 1.776  /B ARG 518 NH2 /A GLU 244 OE1 /B ARG 518 2HH2 3.072 2.220  /B ARG 518 NH2 /A GLU 244 OE2 /B ARG 518 2HH2 3.168 2.221 |
| ###########################################################################  TLR4_0.pdb (Equilibrated snapshot of TLR4/chainA & Vaccine chain C)  7 H-bonds  H-bonds (donor, acceptor, hydrogen, D..A dist, D-H..A dist):  /C LYS 13 NZ /A ASP 50 OD1 /C LYS 13 HZ3 2.831 1.951  /C ARG 92 NE /A GLU 94 OE2 /C ARG 92 HE 2.917 1.985  /C ARG 92 NH2 /A GLU 94 OE1 /C ARG 92 1HH2 2.963 1.993  /C ARG 92 NH2 /A GLU 94 OE2 /C ARG 92 1HH2 3.387 2.686  /C ARG 472 NH1 /A GLU 27 OE1 /C ARG 472 2HH1 2.762 1.786  /C ARG 472 NH2 /A GLU 27 OE1 /C ARG 472 2HH2 3.361 2.673  /C ARG 472 NH2 /A GLU 27 OE2 /C ARG 472 2HH2 2.870 1.874  ###########################################################################  TLR4_last.pdb (Last snapshot of TLR4/chainA & Vaccine chain C)  5 H-bonds  H-bonds (donor, acceptor, hydrogen, D..A dist, D-H..A dist):  /C ARG 92 NE /A GLU 94 OE1 /C ARG 92 HE 3.262 2.398  /C ARG 92 NH2 /A GLU 94 OE1 /C ARG 92 1HH2 3.219 2.285  /C ARG 92 NH2 /A GLU 94 OE2 /C ARG 92 1HH2 2.937 2.083  /C ARG 472 NE /A ASP 50 OD1 /C ARG 472 HE 2.805 1.806  /C ARG 472 NH2 /A ASP 50 OD2 /C ARG 472 1HH2 2.739 1.794  ###########################################################################  No hydrogen bonds found at the interface of TLR4/chainB & Vaccine chain C |
| ###########################################################################  TLR9_0.pdb (Equilibrated snapshot of TLR9/chainA & Vaccine chain B)  14 H-bonds  H-bonds (donor, acceptor, hydrogen, D..A dist, D-H..A dist):  /A ARG 377 NE /B GLU 475 OE2 /A ARG 377 HE 2.791 1.808  /A ARG 377 NH2 /B TYR 15 OH /A ARG 377 2HH2 2.829 1.857  /A ARG 426 NH1 /B TYR 15 O /A ARG 426 2HH1 3.136 2.256  /A ILE 588 N /B TYR 143 OH /A ILE 588 HN 3.127 2.359  /A GLY 612 N /B SER 158 OG /A GLY 612 HN 2.923 2.122  /A ARG 645 NH2 /B THR 279 O /A ARG 645 1HH2 3.568 2.862  /A SER 667 OG /B TYR 71 OH /A SER 667 HG1 3.164 2.224  /A GLN 689 NE2 /B ALA 70 O /A GLN 689 2HE2 3.566 2.677  /B TYR 14 OH /A ASP 424 OD1 /B TYR 14 HH 2.652 1.699  /B SER 158 OG /A ASN 639 O /B SER 158 HG1 2.581 1.724  /B LYS 168 NZ /A ASP 616 OD2 /B LYS 168 HZ1 2.563 1.535  /B ASN 473 ND2 /A MET 400 O /B ASN 473 2HD2 2.899 1.968  /B ARG 514 NH2 /A PHE 375 O /B ARG 514 2HH2 3.257 2.369  /B ARG 518 NH2 /A ARG 346 O /B ARG 518 2HH2 2.658 1.926  ###########################################################################  TLR9_last.pdb  9 H-bonds  H-bonds (donor, acceptor, hydrogen, D..A dist, D-H..A dist):  /A HIS 260 NE2 /B PRO 517 O /A HIS 260 HE2 3.198 2.261  /A ARG 377 NH2 /B GLU 512 O /A ARG 377 1HH2 3.478 2.693  /A ARG 426 NE /B GLU 474 OE1 /A ARG 426 HE 2.703 1.777  /A ARG 426 NH2 /B GLU 474 OE2 /A ARG 426 1HH2 2.816 1.817  /A SER 760 OG /B ALA 70 O /A SER 760 HG1 3.274 2.321  /B LYS 144 NZ /A ASP 587 OD2 /B LYS 144 HZ1 2.707 1.702  /B ARG 472 NE /A ASP 424 OD1 /B ARG 472 HE 2.826 1.873  /B ARG 472 NH2 /A ASP 424 OD1 /B ARG 472 1HH2 2.818 1.907  /B ARG 518 NH2 /A TYR 345 OH /B ARG 518 2HH2 3.489 2.615  ############################################# |

**Supplementary Figures**

**
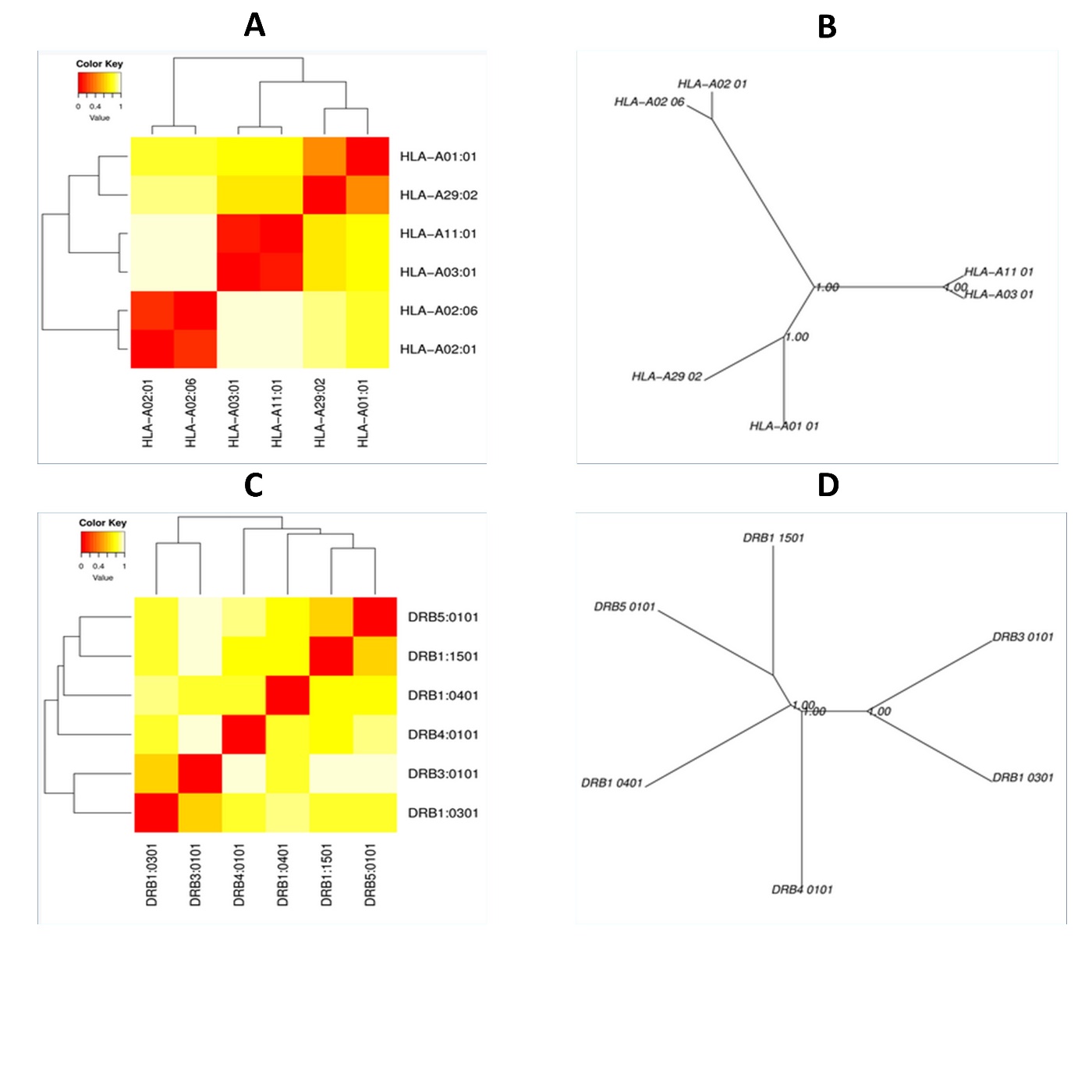
**

**S1 Fig**. The results of the MHC cluster analysis. Here, (A) is the heat map (left) and (B) is the tree map (right) of MHC class-I cluster analysis, (C) is the heat map (left) and (D) is the tree map (right) of MHC class-II cluster analysis.


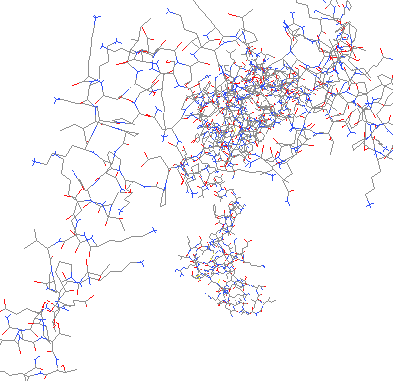


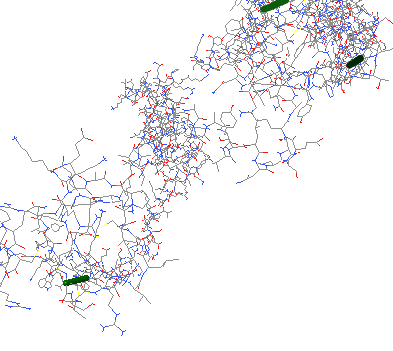


**Original**

**Mutant**

**S2 Fig.** The disulfide engineering of the vaccine construct, both the original (left) and mutant (right) forms are shown.


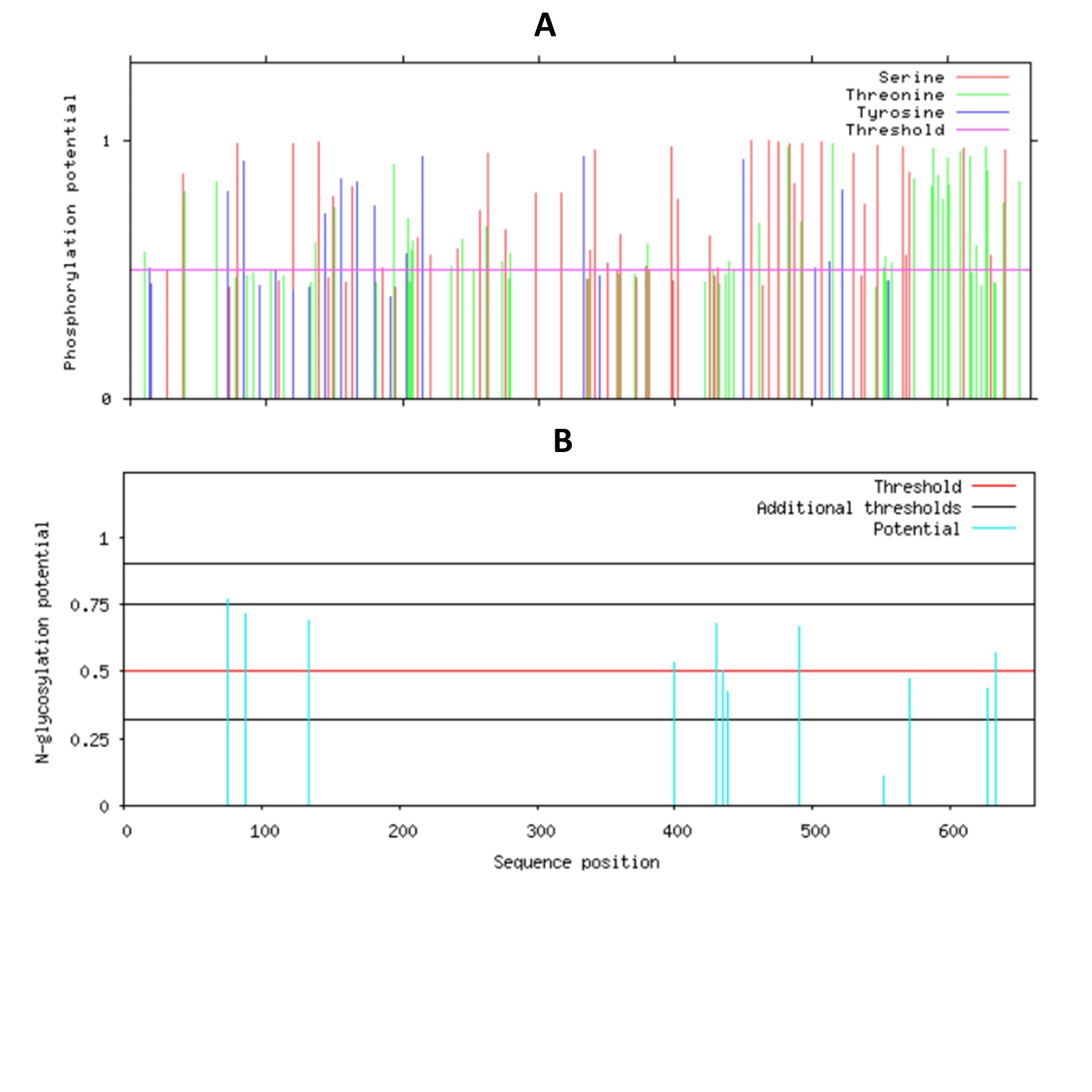


**S3 Fig:** The plots depicting the predicted (A) phosphorylation and (B) N-glycosylation sites in a vaccine construct.

**
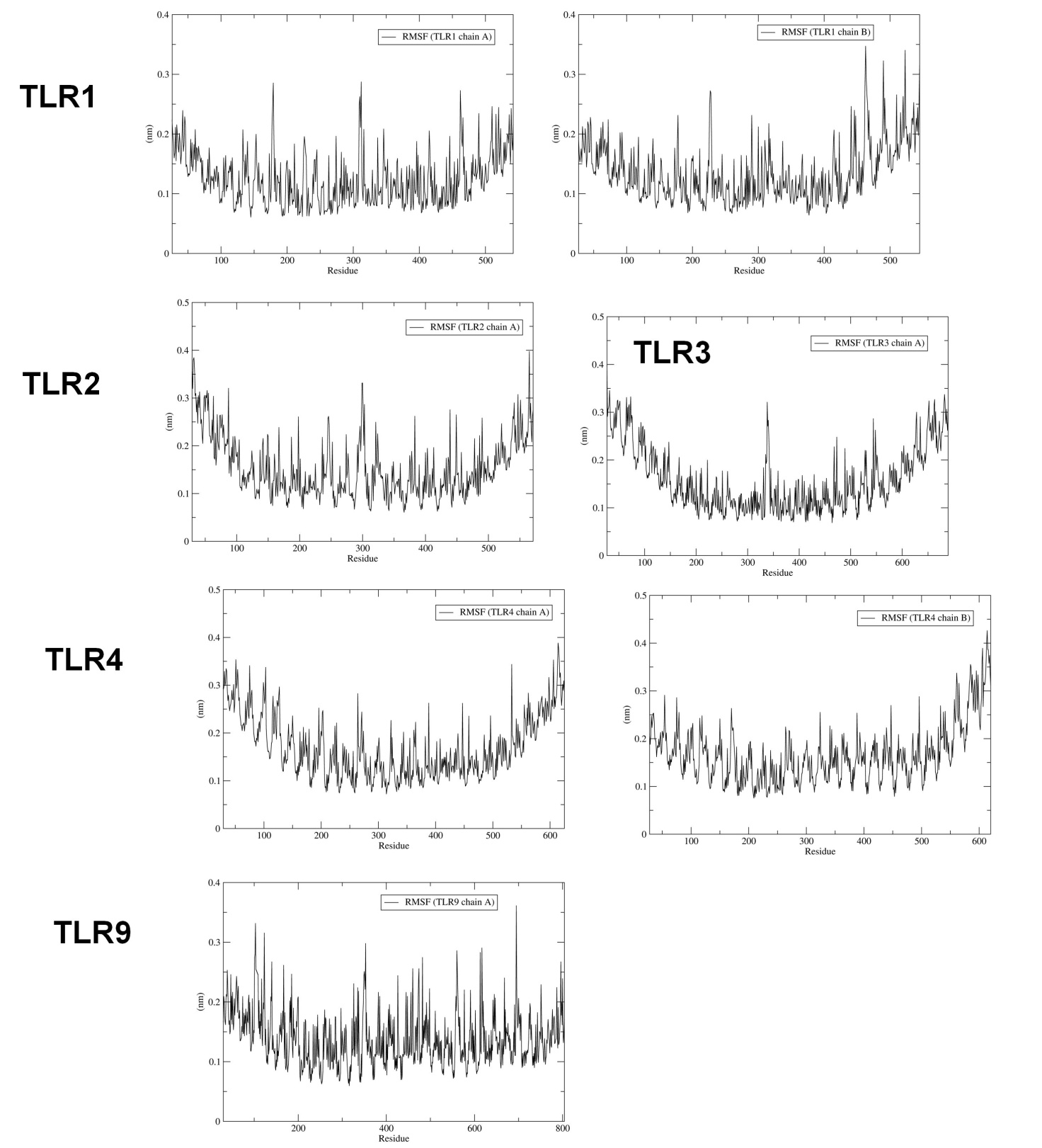
**

**S4 Fig.** RMSF in protein chains in different TLRs.


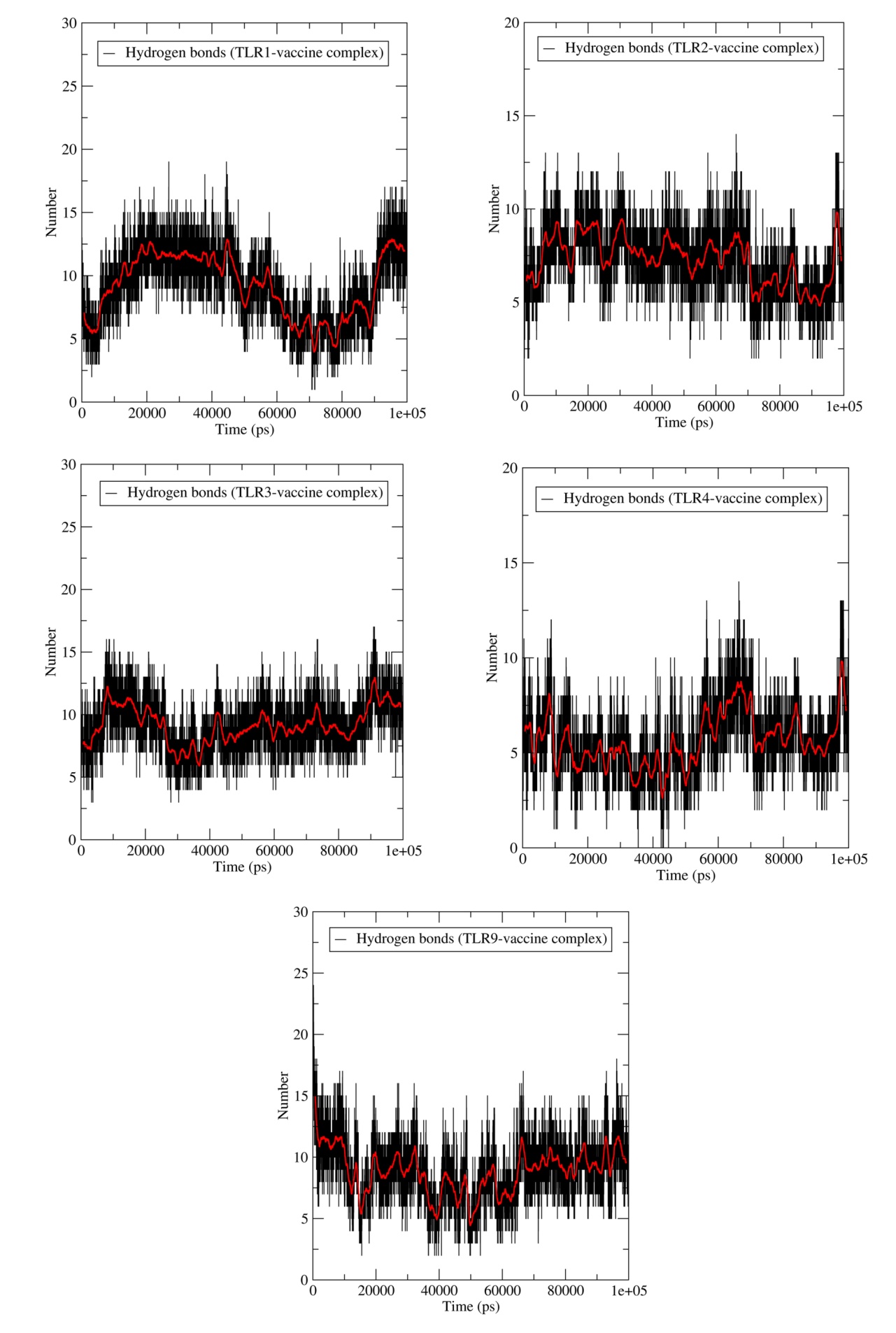


**S5 Fig.** The number of hydrogen bonds formed at the interface of TLR chain and vaccine chain


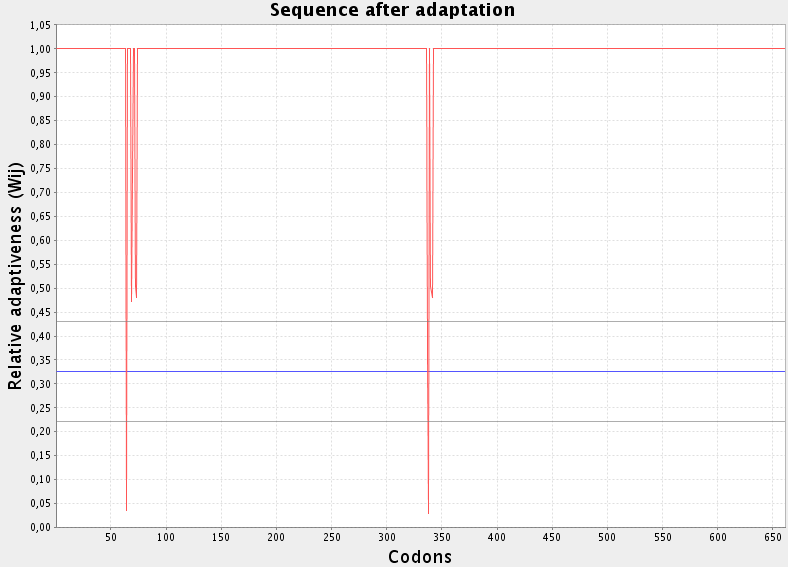


**S6 Fig.** Figure showing the codon adaptation graph of the vaccine.


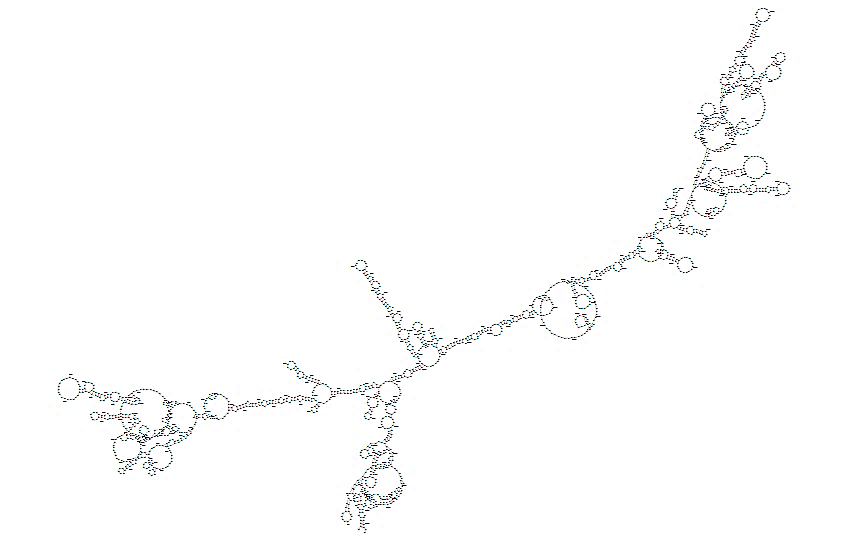


**S7 Fig.** The mRNA secondary structure of the vaccine predicted by the RNAfold server.
